# Identification of Core Genes and Screening of Potential Targets in Intervertebral Disc Degeneration Using Integrated Bioinformatics Analysis

**DOI:** 10.3389/fgene.2022.864100

**Published:** 2022-05-30

**Authors:** Jianjun Li, Cheng Yu, Songjia Ni, Yang Duan

**Affiliations:** ^1^ Department of Spine Surgery, Zhujiang Hospital, Southern Medical University, Guangzhou, China; ^2^ Department of Orthopaedic Trauma, Zhujiang Hospital, Southern Medical University, Guangzhou, China

**Keywords:** intervertebral disc degeneration, differentially expressed genes, hub genes, integrated bioinformatics, protein–protein interaction

## Abstract

**Background:** Intervertebral disc degeneration (IDD), characterized by diverse pathological changes, causes low back pain (LBP). However, prophylactic and delaying treatments for IDD are limited. The aim of our study was to investigate the gene network and biomarkers of IDD and suggest potential therapeutic targets.

**Methods:** Differentially expressed genes (DEGs) associated with IDD were identified by analyzing the mRNA, miRNA, and lncRNA expression profiles of IDD cases from the Gene Expression Omnibus (GEO). The protein–protein interaction (PPI) network, Gene Ontology (GO) enrichment, Kyoto Encyclopedia of Genes and Genomes (KEGG) analysis as well as miRNA–lncRNA–mRNA networks were conducted. Moreover, we obtained 71 hub genes and performed a comprehensive analysis including GO, KEGG, gene set enrichment analysis (GSEA), gene set variation analysis (GSVA), Disease Ontology (DO), methylation analysis, receiver operating characteristic (ROC) curve analysis, immune infiltration analysis, and potential drug identification. We finally used qRT-PCR to verify 13 significant DEGs in normal and degenerative nucleus pulposus cells (NPCs).

**Results:** We identified 305 DEGs closely related to IDD. The GO and KEGG analyses indicated that changes in IDD are significantly associated with enrichment of the inflammatory and immune response. GSEA analysis suggested that cell activation involved in the inflammatory immune response amide biosynthetic process was the key for the development of IDD. The GSVA suggested that DNA repair, oxidative phosphorylation, peroxisome, IL-6-JAK-STAT3 signaling, and apoptosis were crucial in the development of IDD. Among the 71 hub genes, the methylation levels of 11 genes were increased in IDD. A total of twenty genes showed a high functional similarity and diagnostic value in IDD. The result of the immune cell infiltration analysis indicated that seven genes were closely related to active natural killer cells. The most relevant targeted hub genes for potential drug or molecular compounds were MET and PIK3CD. Also, qRT-PCR results showed that ARHGAP27, C15orf39, DEPDC1, DHRSX, MGAM, SLC11A1, SMC4, and LINC00887 were significantly downregulated in degenerative NPCs; H19, LINC00685, mir-185-5p, and mir-4306 were upregulated in degenerative NPCs; and the expression level of mir-663a did not change significantly in normal and degenerative NPCs.

**Conclusion:** Our findings may provide new insights into the functional characteristics and mechanism of IDD and aid the development of IDD therapeutics.

## Introduction

Low back pain (LBP) is one of the most common musculoskeletal disorders and has been estimated to affect 60–80% of adults (Wu et al., 2021). LBP represents a heavy burden for patients as well as the society (Liu et al., 2021) and leads to disability (Zhang et al., 2021). Intervertebral disc degeneration (IDD) is recognized as the major cause of LBP (Smolders et al., 2013). Current treatment of IDD, including conservative therapies and surgical interventions, often only alleviates the patients’ pain without preventing the progression of IDD (Stich et al., 2020). Furthermore, the clinical results are suboptimal (Wang H. et al., 2020). Surgical therapies are generally associated with various complications (Smolders et al., 2013). Therefore, a comprehensive understanding of the pathological changes and molecular mechanisms underlying IDD appears to be essential for diagnosis and developing new therapeutic approaches.

Currently, the research on mechanisms of action of IDD mainly focuses on tissue fibrosis (Wang X. et al., 2019), inflammatory responses (Chang et al., 2021), the anabolism of extracellular matrix (Wang R. et al., 2020), insufficient transport of metabolites (Ge et al., 2020), enhanced aggrecan and collagen degradation (Du et al., 2021), and the structural and functional abnormalities in mitochondria (Song et al., 2021). However, the exact mechanisms of IDD remain elusive as the pathogenesis of IDD is complex and heterogeneous. With the rapid and extensive use of high-throughput system technologies, integrated bioinformatics analysis has emerged as a promising approach to explore the mechanism of IDD.

In this study, we identified differentially expressed genes (DEGs) associated with IDD by analyzing two mRNA expression profiles, three miRNA expression profiles, and two long non-coding (lnc) RNA expression profiles that were downloaded from the GEO database. Next, we constructed a PPI network and performed Gene Ontology (GO) and Kyoto Encyclopedia of Genes and Genomes (KEGG) enrichment analyses of the DEGs from the PPI network. Subsequently, we constructed the lncRNA–miRNA–mRNA network. We obtained 71 differentially co-expressed mRNAs and performed a comprehensive analysis on them. GO, KEGG, gene set enrichment analysis (GSEA), and gene set variation analysis (GSVA) were used to study the molecular mechanisms of IDD. The Disease Ontology (DO) was used to study the association between genes and diseases. Methylation levels were validated by GSE129789. Diagnostic accuracy was assessed using a receiver operating characteristic (ROC) curve analysis. The immune infiltration was analyzed using the CIBERSORT algorithm. We then predicted potential drugs or molecular compounds using the DGIdb database, and finally we used qRT-PCR to verify the 13 significant DEGs in normal and degenerative nucleus pulposus cells (NPCs). Our findings regarding the mechanisms of IDD could be used for further exploration of biomarkers or treatment targets for IDD.

## Materials and Methods

### Microarray Data

The Gene Expression Omnibus (GEO) database (http://www.ncbi.nlm.nih.gov/geo) (Barrett et al., 2007) is a public database of high-throughput gene expression data and microarrays. In total, seven gene expression datasets were derived from GEO, and each dataset was divided into healthy and IDD subgroups (Table 1). GSE124272 (Wang Y. et al., 2019) and GSE150408 (Wang et al., 2021) are both series of the GPL21185 Agilent-072363 SurePrint G3 Human GE v3 8 × 60 K Microarray 039494. GSE63492 (Liu et al., 2015), GSE16726 (Ji et al., 2018), GSE19943 (Wang et al., 2011), GSE56081 (Wan et al., 2014), GSE153761, and GSE129789 are series of the GPL19449 Exiqon miRCURY LNA microRNA Array, seventh generation REV-hsa, mmu, & rno (miRBasev18.0), GPL8701Agilent *Escherichia coli* 15 K array, GPL9946 Exiqon human miRCURY LNA™ microRNA Array V11.0, GPL15314 Arraystar Human LncRNA microarray V2.0 (Agilent-033010 Probe Name version), GPL22120 Agilent-078298 human ceRNA array V1.0 4 × 180 K, and GPL21145 Infinium MethylationEPIC. The data type was microarray expression profiles, and the species selected was *Homo sapiens*.

**TABLE 1:** Information of data.

GEO	Normal	Intervertebral disc degeneration
GSE124272	8	8
GSE150408	17	17
GSE63492	5	5
GSE116726	3	3
GSE19943	3	3
GSE56081	5	5
GSE153761	3	3
GSE129789	8	8

### Identification of DEGs and Hub Genes

First, we performed data normalization. Next, DEGs were screened using the “limma” packages (Ritchie et al., 2015). The genes with |log (FC)|> 1 and *p*-value < 0.05 were defined as differentially expressed genes. Subsequently, the volcano plotting tool (http://soft.sangerbox.com/), “pheatmap” package in R software, and Online tool Draw Venn Diagram (http://bioinformatics.psb.ugent.be/webtools/Venn/) were used to plot the volcano maps, the heatmap, and the Venn map of DEGs. We obtained hub genes associated with IDD by intersecting differentially expressed mRNA with target genes of differentially expressed miRNAs.

### Protein–Protein Interaction Network Construction

The STRING database (Szklarczyk et al., 2019) contains known protein and predicted protein interactions and includes results from experimental data, results from text mining of PubMed abstracts, and results from other databases as well as results predicted using bioinformatics methods. We used the STRING database to select differentially expressed genes with a combined score of more than 400 to construct a mRNA-related protein–protein interaction network (PPI) and visualized the network model using Cytoscape (version 3.6.1) (Shannon et al., 2003). The DEGs were functionally annotated using “clueGO” (Bindea et al., 2009).

### Functional Enrichment Analyses of DEGs and Hub Genes

GO functional annotation analysis is a common method for large-scale functional enrichment research of genes, including biological process (BP), molecular function (MF), and cellular component (CC) (Ashburner et al., 2000). KEGG is a widely used database for storing information about genomes, biological pathways, diseases, and drugs (Kanehisa and Goto, 2000). We used the “clusterProfiler” of the R package (Yu et al., 2012) to analyze the function of DEGs from the PPI network and hub genes.

### Disease Ontology

DO allows the enrichment analysis of diseases according to genes, and the similarities between diseases play an important role in studying and understanding the pathogenesis of complex diseases, early prevention and diagnosis of critical diseases, research and development of new drugs, and drug safety evaluation. We used “DOSE” of R package (Ashburner et al., 2000) to analyze the functions of hub genes.

### lncRNA–miRNA–mRNA Network

MiRNA is a type of endogenously encoded non-coding single-stranded RNA molecule, with a length of 19–25 nucleotides. Generally, miRNAs have a complex regulatory network. One miRNA can regulate multiple target genes, and the same target gene can be regulated by multiple miRNAs (Lu and Rothenberg, 2018). LncRNA is a type of RNA molecule with a transcript length of more than 200 nucleotides. It is generally agreed that they do not encode proteins but rather participate in the epigenetic, transcriptional, and post-transcriptional regulation of protein-encoding genes (Arraiano, 2021). We obtained the differentially expressed miRNA-related mRNA and lncRNA from the miRNet database (Fan and Xia, 2018) and visualized the miRNA–mRNA–lncRNA regulatory network using Cytoscape software.

### Gene Set Enrichment Analysis of Hub Genes

We used GSEA to evaluate the distribution trend of genes in a predefined gene set sorted by the degree of phenotypic correlation to judge the contribution to the specific phenotype (Subramanian et al., 2005). The “c2.all.v7.4.symbols” and “c5.all.v7.4.symbols” obtained from the MSigDB database were selected as the reference gene sets (Liberzon et al., 2015).

### Gene Set Variation Analysis of Hub Genes

We used GSVA which is a non-parametric and unsupervised analysis method that was mainly used to evaluate the gene set enrichment results of on-chip nuclear transcriptome by transforming the gene expression matrix between different samples into the gene set expression matrix between samples (Wang et al., 2011). The “h.all.v7.4.symbols.gmt” gene set obtained from the MSigDB database was selected as the reference gene set.

### Methylation Analysis of Hub Genes

GSE129789, the genome-wide DNA methylation profile of human IDD, which has series on GPL21145 Infinium MethylationEPIC, was used to annotate the methylation levels of hub genes in IDD.

### ROC Analysis and Friend Analysis of Hub Genes

ROC curve analysis, which yields indicators of accuracy such as the area under the curve (AUC), provides the fundamental principle and rationale for distinguishing between the specificity and sensitivity of diagnostic performance. We used the pROC package of R software (Robin et al., 2011) to conduct our ROC curve analysis. To analyze the functional correlation between hub genes, we used the GOSemSim package of R software (Yu, 2020) for friend analysis of hub genes.

### Immune Infiltration Analysis and ROC Analysis

CIBERSORT is an algorithm that deconvolutes the expression matrix of immune cell subtypes based on the linear support vector regression principles. We used RNA-Seq data (Newman et al., 2019) and CIBERSORT algorithm for immune infiltration analysis. Next, we selected the meaningful immune cells which were divergent between the two datasets and used the ROC curve analysis to analyze the correlation between hub genes and meaningful immune cells.

### Potential Drug Identification

We used DGIdb version 3.0.2 (https://www.dgidb.org), a public resource for drug targeting and sensitive genomes and drug–gene interactions (Cotto et al., 2018), to predict potential drugs or molecular compounds interacting with DEGs and used Cytoscape software to visualized the drug–hub gene interaction network.

### Nucleus Pulposus Cell Isolation and Culture

Normal intervertebral disc tissue was collected from a patient with organ donation. Human NP tissue was cut into pieces and digested in 0.2% type II collagenase (Thermo Fisher Scientific, Massachusetts, United States) and 0.25% trypsin (Thermo Fisher Scientific) for 3 h. After filtration and washing with PBS, the suspension was centrifuged, and the isolated cells were cultured in DMEM containing 15% fetal bovine serum (FBS, Thermo Fisher Scientific) and 1% penicillin–streptomycin (Thermo Fisher Scientific). The medium was changed twice a week, and the NPCs of the third generation were used in the following experiments. The experimental scheme was approved by the Ethics Committee of Zhujiang Hospital of Southern Medical University.

### qRT-PCR

The third generation NPCs were divided into the normal group and degenerative group. The cells in the degenerative group were treated with the TNF-α (100 ng/ml) culture medium, the cells were collected after 48 h, and then used for follow-up experiments. The total RNA of NPCs was extracted and synthesized into cDNA according to the manufacturer’s protocol (Promega Biotech Co., Ltd, Beijing, China). qRT-PCR was performed on a LightCycler 96 (Roche Life Sciences, Switzerland, Basel) using Real-Time PCR Mix (Vazyme Biotech, Nanjing, Jiangsu Province, China). The gene expression relative to GAPDH and U6 expression was assessed using the 2^−ΔΔCt^ method (Xiang et al., 2020). Independent experiments were conducted in triplicate (the sequence fragments of RNAs are shown in Table 2).

**TABLE 2:** Sequence fragments of RNAs.

ARHGAP27	ACC​ACC​TGG​GAG​TCG​CCC​TT	TCC​TCA​TCC​CAG​TAC​TGG​CC
C15orf39	TCC​AGC​ATC​TAC​TTC​AGC​CC	TCT​AGC​CAC​TCG​CGA​AGC​TT
DEPDC1	ATG​TGT​GTT​ATG​CTG​TGC​TG	AGG​GTA​CTT​GAA​GAA​TTT​C
DHRSX	GGT​CAC​CGT​CTC​CTC​TGC​CA	GCG​TAG​GCT​GCG​TGG​GGT​GA
MGAM	TCG​ACT​GCC​TAG​CAC​TAA​CG	TTG​GGA​GTT​GTG​TCT​CTG​T
SLC11A1	GCT​TCT​TCG​GGG​CCT​GTT​CC	AGA​CTT​GAC​CAG​GGC​CGA​GT
SMC4	CAT​TTA​AGG​ATG​TTG​GAA​AT	CAT​ACC​CTC​ATC​GTG​TTC​AG
LINC00887	ATG​AAT​TGG​TGT​CTA​TAT​TG	AGG​GTA​AAT​TCT​GGT​GAA​C
H19	CAC​AGG​ACA​GAG​GGG​TCC​CC	TGT​CTT​CCC​GCT​CTC​CAG​C
LINC00685	CTC​GGG​AGC​CCC​TGC​GGT​GG	TCT​TAC​TGT​CCC​CTG​GAC​CA
hsa-mir-185-5p	ACA​CTC​CAG​CTG​GGT​GGA​GAG​AAA​GGC​AGT​T	CTCAACTGGTGTCGTGGA
hsa-mir-4306	ACA​CTC​CAG​CTG​GGC​CTT​AGA​GTC​TCC​AGA​G	CTCAACTGGTGTCGTGGA
hsa-mir-663a	ACA​CTC​CAG​CTG​GGT​CCC​AGG​CGG​GGC​GCC​GC	CTCAACTGGTGTCGTGGA
U6	CTCGCTTCGGCAGCACA	AAC​GCT​TCA​CGA​ATT​TGC​GT
GAPDH	GCT​CAT​TTG​CAG​GGG​GGA​G	GTT​GGT​GGT​GCA​GGA​GGC​A

### Statistical Analysis

All data calculations and statistical analyses were performed using R programming (https://www.r-projec t.org/, version 4.0.2). Multiple test corrections were performed using Benjamini I–Hochberg and false discovery rate to reduce the false-positive rate. Between groups, comparisons were performed using the independent Student *t*-test. The non-normally distributed variables were analyzed using Mann–Whitney *U* tests. Two-tailed *p*-values < 0.05 were considered statistically significant.

## Results

### Identification of DEGs

**FIGURE 1:**
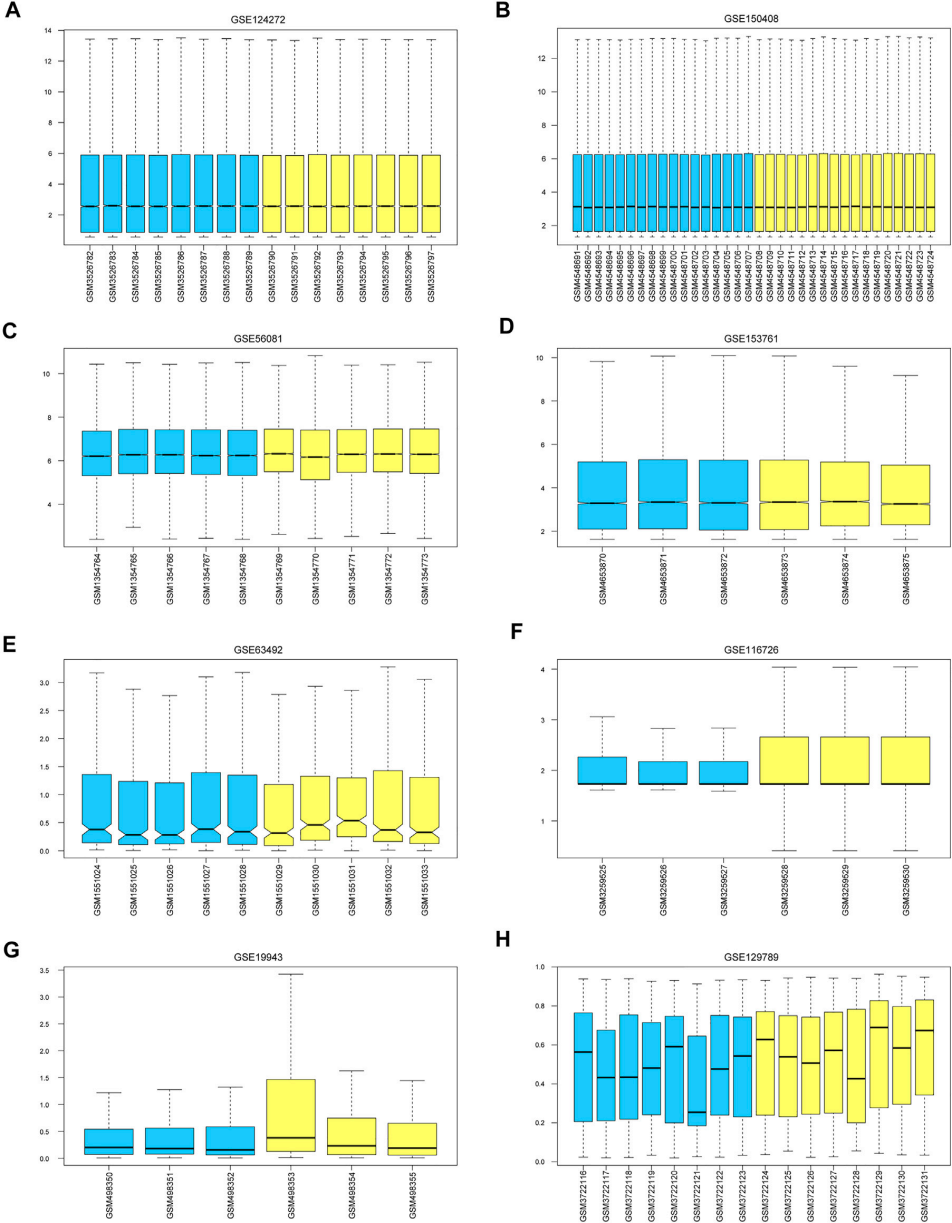
Calibration of eight sets of data. The blue color represents the degenerative intervertebral disc tissue, and the yellow color represents the normal intervertebral disc tissue. We marked **(A–H)** represent the Calibration of eight sets of data.

**FIGURE 2:**
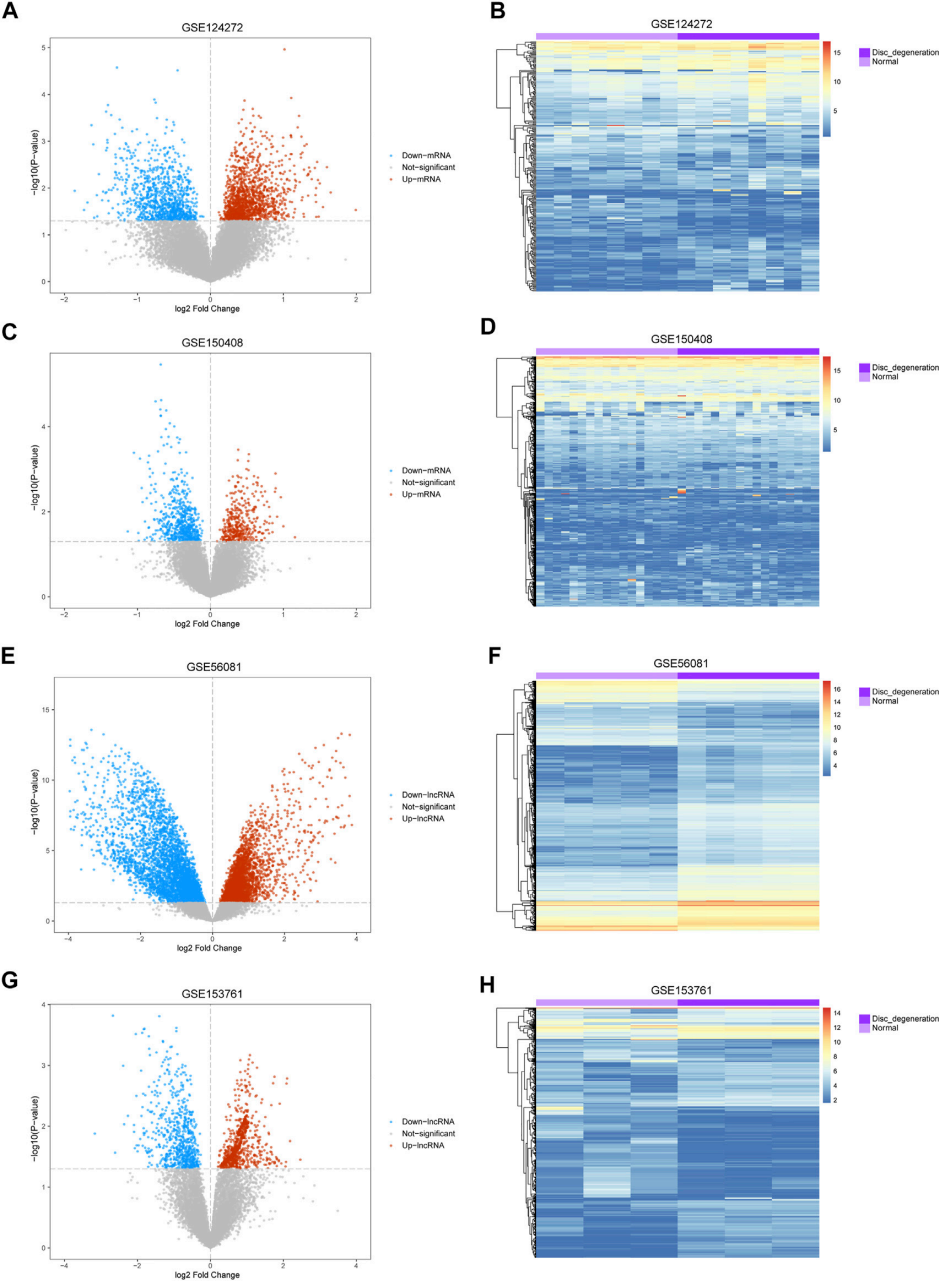
Differential expression of mRNA and lncRNA associated with IDD. **(A,C,E,G)** Volcano map of differentially expressed genes. The abscissa represents the log2-fold change, and the ordinate represents the -log_10_ (*p*-value). The red node indicates the upregulated differentially expressed genes, the blue node indicates the downregulated differentially expressed genes, and the gray node indicates genes that are not significantly differentially expressed. **(B,D,F,H)** Heatmap of differentially expressed genes. The abscissa indicates the patient ID, and the ordinate indicates the respective differentially expressed genes. Red represents the high gene expression, and blue represents the low gene expression. The dark purple annotation bar represents the degenerative intervertebral disc tissue, and the light purple annotation bar represents the normal intervertebral disc tissue.

**FIGURE 3:**
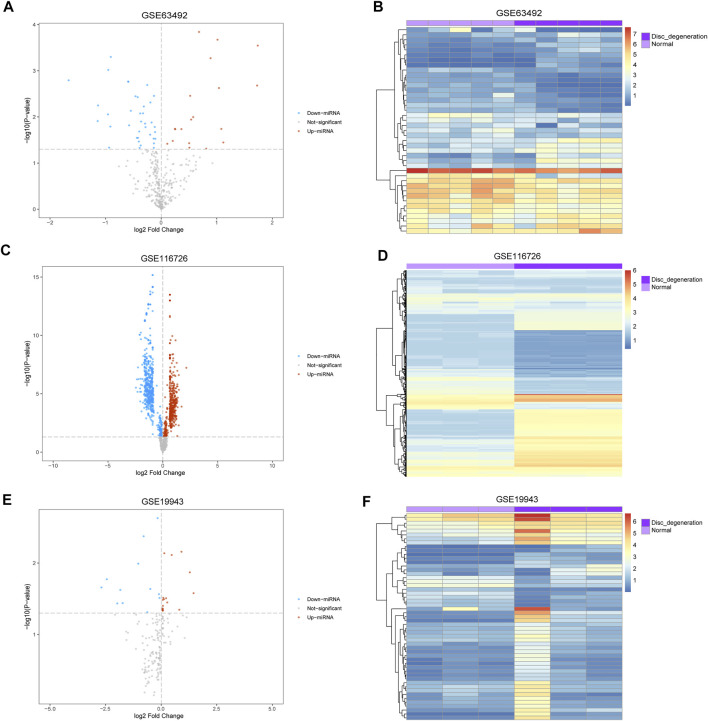
Differentially expressed miRNA associated with disc degeneration. **(A,C,E)** Volcano map of differentially expressed genes. The abscissa represents log2-fold Change, and the ordinate represents -log10 (*p*-value). The red node indicates upregulated differentially expressed genes, the blue node indicates downregulated differentially expressed genes, and the gray node indicates genes that are not significantly differentially expressed. **(B,D,F)** Heatmap of differentially expressed genes. The abscissa represents the patient ID, and the ordinate represents the respective differentially expressed genes with red indicating high gene expression and blue indicating low gene expression. The dark purple comment bar indicates the degenerative disc tissue, and the light purple comment bar indicates the normal disc tissue.

**FIGURE 4:**
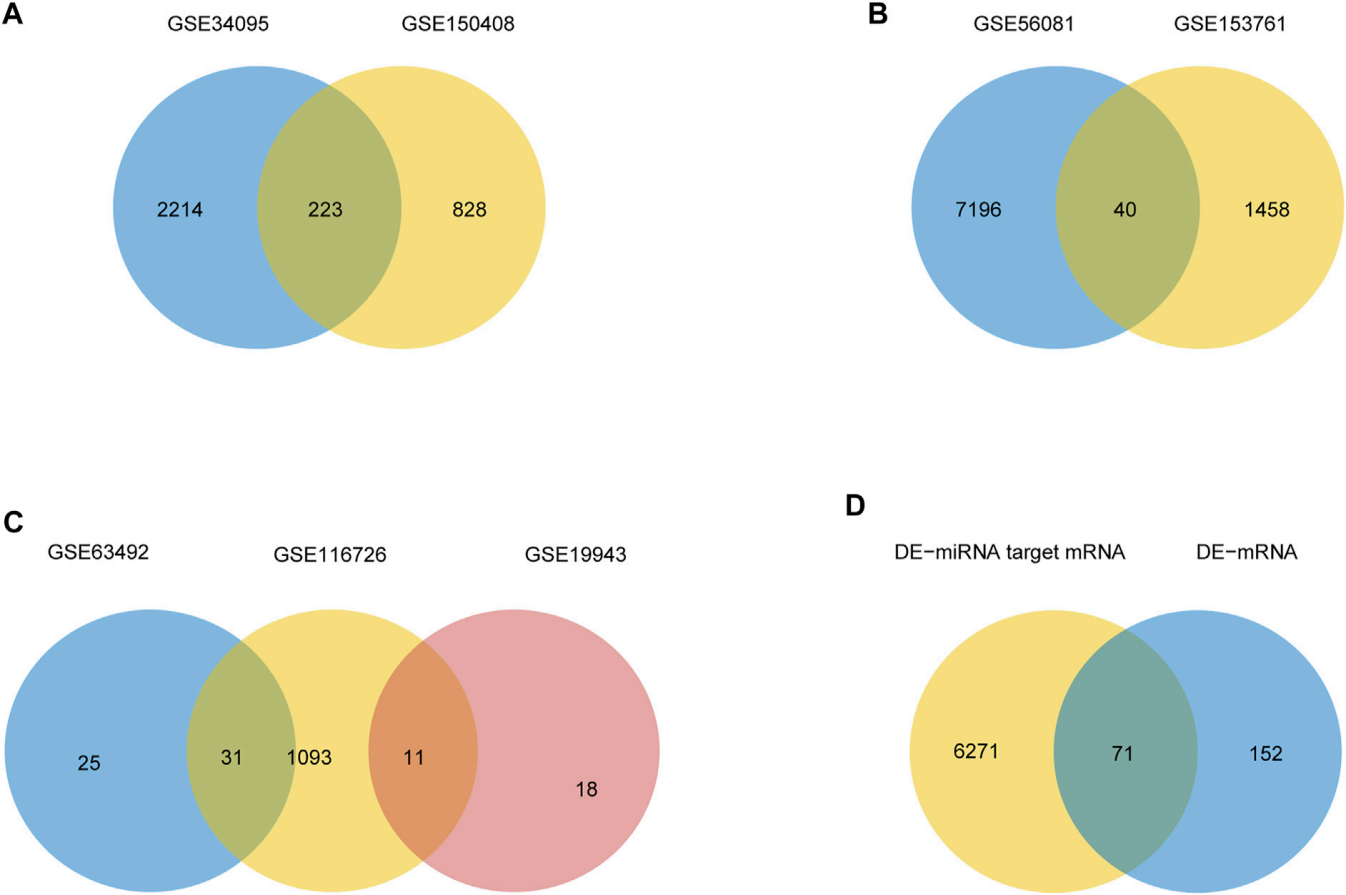
Differential expression of mRNA, lncRNA, miRNA, and hub gene in IDD. **(A)** Venn diagram of differentially expressed genes in two sets of mRNA data. **(B)** Venn diagrams of differentially expressed genes in two sets of lncRNA datasets. **(C)** Venn diagrams of differentially expressed genes in three sets of miRNA data. **(D)** IDD-related differentially expressed mRNA and miRNA-targeted mRNA Venn diagram.

The mean values of gene expression for each dataset were approximately the same after normalization (Figure 1). Following the standardization of the microarray results, 1,125 upregulated genes and 2,831 downregulated genes were identified in seven microarray datasets; we also performed cluster analysis on the expression values of DEGs, and the results showed that DEGs could well distinguish between degenerative disc tissue and normal disc tissue (Figures 2, 3). Furthermore, overlap of DEGs (223 overlapping mRNAs in GSE124272 and GSE150408; 40 overlapping lncRNAs in GSE56081 and GSE153761; and 42 overlapping miRNAs in GSE63492, GSE116726, and GSE19943) was identified (Figures 4A–C). We identified 223 differentially expressed mRNAs and found 6,342 target genes regulated by 42 differentially expressed miRNAs. A total of 71 hub genes related to disc degeneration were obtained by the intersection of the 223 differentially expressed mRNAs and 6,342 target genes (Figure 4D; Table 3).

**TABLE 3:** Total 71 hub genes related to disc degeneration.

ETV6	TMOD3	KLF4	GSN	EVC2	FKBP1A	FAM160B1	ZNF595
KIAA0930	FNBP1L	ZFP36L2	IL2RB	RAP1B	TCFL5	ARHGAP27	—
SIGLEC9	CD46	ACVR2B	SERPINA1	SON	HNRNPL	C15orf39	
PLEKHO1	SSH2	ATG2A	RARA	WWP2	MET	DHRSX	
GLCCI1	INAFM1	C5AR2	SLC11A1	LTF	SMC4	AFF1	
OCIAD2	CREBRF	ZNF286A	TMEM127	DEPDC1	DPH5	TPRN	
KCNRG	MAN1A2	MYO1F	PHC2	ZFR	ZSWIM6	MEX3C	
NCF1	NOA1	STAT5A	CSNK2A1	KIAA1107	SORT1	MTF1	
KHSRP	RNF19B	LEPROTL1	PLIN5	NUCB2	LYST	ZNF567	
HNRNPA0	PIK3CD	ATP6V1C1	MGAM	FRY	C9orf72	AKR1C3	

### PPI Network

**FIGURE 5:**
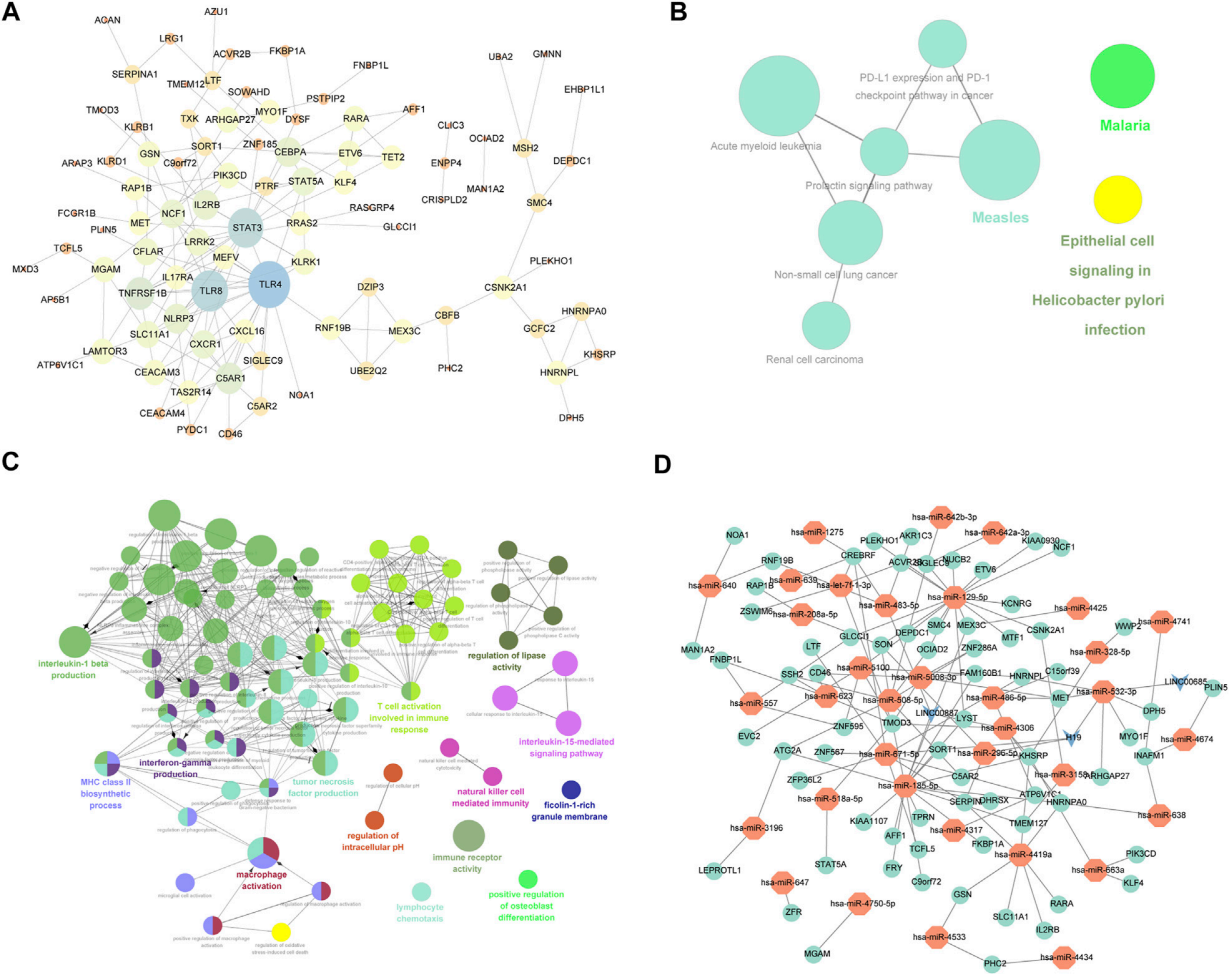
mRNA-related PPI networks and ceRNA networks differentially expressed in IDD. **(A)** Protein interaction network of differentially expressed mRNA associated with disc degeneration. **(B)** clueGO-GO analysis of genes in PPIs that differentially express mRNAs associated with disc degeneration. **(C)** clueGO-KEGG analysis of genes in PPIs that differentially express mRNAs associated with disc degeneration. **(D)** ceRNA network of hub genes. The red node represents the hub gene, the green node represents the miRNA that regulated the hub gene, and the blue node represents lncRNA.

The interactions of 223 differentially expressed mRNAs were analyzed using the STRING online database to investigate the PPI network underlying IDD. The network model was visualized using Cytoscape (version 3.6.1). The network contains 93 genes and 163 interaction pairs. The average degree of nodes is 2.88, and the local clustering coefficient is 0.518. TLR4 interacted with 20 differentially expressed mRNAs, TLR8 interacted with 16 differentially expressed mRNAs, and STAT3 interacted with 15 differentially expressed mRNAs (Figure 5A).

### GO and KEGG Enrichment Analyses of DEGs

To verify the function of differentially expressed mRNA in IDD, we first performed GO and KEGG on DEGs obtained from the PPI network. GO analysis results showed that changes in BPs of DEGs were mainly enriched in T-cell activation involved in the immune response, macrophage activation, positive regulation of interleukin (IL)-5 production, regulation of lipase activity, negative regulation of cysteine-type endopeptidase activity, IL-15-mediated signaling pathway, lymphocyte chemotaxis, positive regulation of osteoblast differentiation, and regulation of IL-1 beta production (Figure 5B). The result of KEGG pathway enrichment indicated that DEGs were significantly enriched in epithelial cell signaling following *Helicobacter pylori* infection, renal cell carcinoma, malaria, measles, and prolactin signaling pathway (Figure 5C).

### lncRNA–miRNA–mRNA Network

The top 3 targeted DEGs for miRNAs were TAOK1 (modulated by 12 miRNAs), G3BP1 (modulated by 11 miRNAs), and GIGYF1 (modulated by 11 miRNAs). The top 3 DEGs that may control the mRNA copy number were hsa-mir-185-5p that controlled 1,142 mRNAs, hsa-mir-129-5p that controlled 1,111 mRNAs, and hsa-mir-671-5p that controlled 1,081 mRNAs. The top 3 targeted differentially expressed lncRNAs for miRNAs were NEAT1 (modulated by 13 miRNAs), KCNQ10T1 (modulated by 10 miRNAs), and LINC00963 (modulated by 10 miRNAs). The top 3 miRNAs that may control the lncRNA copy number were hsa-mir-185-5p that controlled 66 lncRNAs, hsa-mir-4306 that controlled 66 lncRNAs, and hsa-let-7f-5p that controlled 53 lncRNAs. Also, we constructed a miRNA–mRNA–lncRNA regulatory network between differentially expressed mRNAs, miRNAs, and lncRNAs (Figure 5D), which contained 71 mRNAs, 35 miRNAs, and 3 lncRNAs. The first 2 mRNAs regulated by the most miRNAs are TMOD3 regulated by 7 miRNAs and KHSRP regulated by 6 miRNAs. The first 2 miRNAs controlling multiple genes are hsa-mir-129-5p and hsa-mir-185-5p, which control 17 genes, respectively. (Figure 5D).

### GO, KEGG, and DO Enrichment Analyses of Hub Genes

**FIGURE 6:**
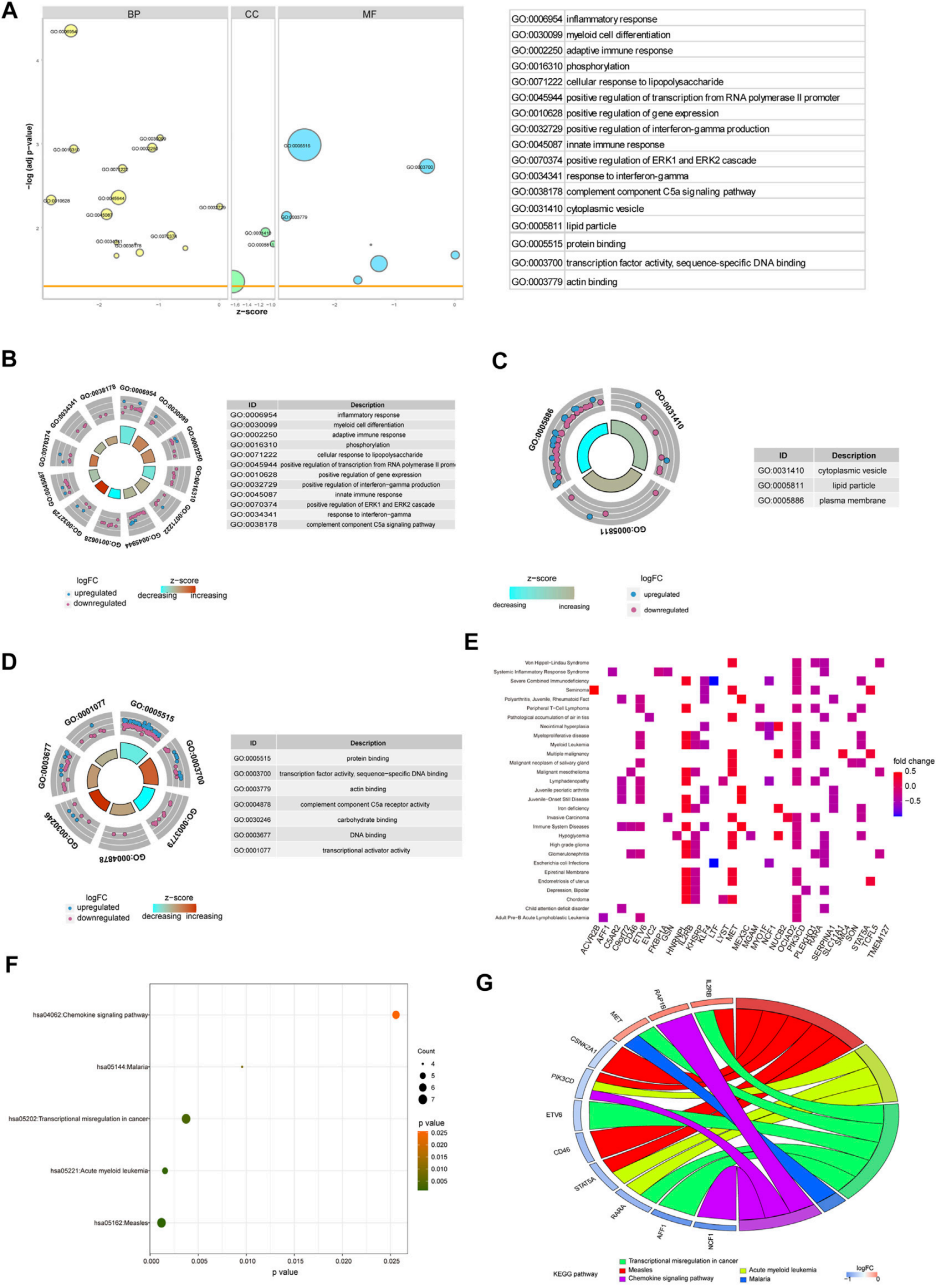
Enrichment analysis of hub genes. **(A)** GO enrichment analysis of hub genes. **(B–D)** BP, CC, and MF for enrichment analysis. Gene expression display of the first 12 results of BP, the first three results of CC, and the first eight results of MF in enrichment analysis. The node color indicates the gene expression level, purple indicates the upregulation of gene expression, and blue indicates the downregulation of gene expression. **(E)** DO enrichment analysis of hub genes. The horizontal axis is the gene name, and the vertical axis is the disease name. Color indicates gene expression level, red indicates gene upregulation, and blue indicates gene downregulation. **(F)** KEGG enrichment analysis results of hub genes, in which node color indicates the significance level of pathway enrichment and node size indicates the number of genes enriched in the pathway. **(G)** For the first five enrichment results in KEGG enrichment analysis, the rectangular color indicates the gene expression level, red indicates the upregulation of gene expression, and blue indicates the downregulation of gene expression. Line colors identify different pathways.

To verify the function of the 71 hub genes, we performed GO function annotation (Figure 6A). The GO analysis showed that changes in the BPs of hub genes were mainly enriched in the following biological processes: inflammatory response, myeloid cell differentiation, adaptive immune response, phosphorylation, cellular response to lipopolysaccharide, positive regulation of transcription from RNA polymerase II promoter, positive regulation of gene expression, positive regulation of interferon-gamma production, innate immune response, and positive regulation of ERK1 and ERK2 cascade (Figure 6B). The changes in the CCs of hub genes were significantly enriched in cytoplasmic vesicles, lipid particles, and the plasma membrane (Figure 6C). The changes in the MFs of hub genes were significantly enriched in protein binding, transcription factor activity, sequence-specific DNA binding, actin binding, complement component C5a receptor activity, carbohydrate binding, DNA binding, transcriptional activator activity, and RNA polymerase II core promoter proximal region sequence-specific binding (Figure 6D). The result of DO analysis indicated that hub genes were significantly enriched in myeloproliferative disease, acute leukemia, malignant neoplasm of the salivary gland, *Escherichia coli* infections, immunologic deficiency syndromes, epiretinal membrane, chronic myeloproliferative disorder, malignant mesothelioma, polycythemia vera, and familial dementia (Figure 6E). The KEGG pathway analysis indicated that hub genes were mainly enriched in measles, acute myeloid leukemia, transcriptional mis-regulation in cancer, malaria, and chemokine signaling pathway (Figure 6F). We demonstrated gene expression in these five pathways (Figure 6G). Also, the functional annotation of hub genes is shown in Table 4.

**TABLE 4:** Functional annotation of hub genes.

Ontology	ID	Description	p.adjust
BP	GO:0006954	Inflammatory response	4.49666E-05
BP	GO:0030099	Myeloid cell differentiation	0.000851612
BP	GO:0002250	Adaptive immune response	0.001115243
BP	GO:0016310	Phosphorylation	0.001153013
BP	GO:0071222	Cellular response to lipopolysaccharide	0.001982371
BP	GO:0045944	Positive regulation of transcription from RNA polymerase II promoter	0.004375852
BP	GO:0010628	Positive regulation of gene expression	0.004689123
BP	GO:0032729	Positive regulation of interferon-gamma production	0.005632371
BP	GO:0045087	Innate immune response	0.006927918
BP	GO:0070374	Positive regulation of ERK1 and ERK2 cascade	0.012421465
CC	GO:0031410	Cytoplasmic vesicle	0.011367083
CC	GO:0005811	Lipid particle	0.015671129
CC	GO:0005886	Plasma membrane	0.044085504
MF	GO:0005515	Protein binding	0.001033583
MF	GO:0003700	Transcription factor activity and sequence-specific DNA binding	0.001857828
MF	GO:0003779	Actin binding	0.007338964
MF	GO:0004878	Complement component C5a receptor activity	0.016048357
MF	GO:0030246	Carbohydrate binding	0.021283222
MF	GO:0003677	DNA binding	0.026951136
MF	GO:0001077	Transcriptional activator activity and RNA polymerase II core promoter proximal region sequence-specific binding	0.042305005
KEGG	hsa05162	Measles	0.001180685
KEGG	hsa05221	Acute myeloid leukemia	0.001547923
KEGG	hsa05202	Transcriptional misregulation in cancer	0.003738135
KEGG	hsa05144	Malaria	0.00956855
KEGG	hsa04062	Chemokine signaling pathway	0.025573188
DO	umls:C0027022	Myeloproliferative disease	2.4474E-05
DO	umls:C0085669	Acute leukemia	4.63915E-05
DO	umls:C0220636	Malignant neoplasm of the salivary gland	7.27483E-05
DO	umls:C0014836	*Escherichia coli* infections	0.00010599
DO	umls:C0021051	Immunologic deficiency syndromes	0.000131517
DO	umls:C0339543	Epiretinal membrane	0.000199137
DO	umls:C1292778	Chronic myeloproliferative disorder	0.000255639
DO	umls:C0345967	Malignant mesothelioma	0.000364185
DO	umls:C0032463	Polycythemia vera	0.000525201
DO	umls:C0751071	Familial dementia	0.000553109

### GSEA Analysis

**FIGURE 7:**
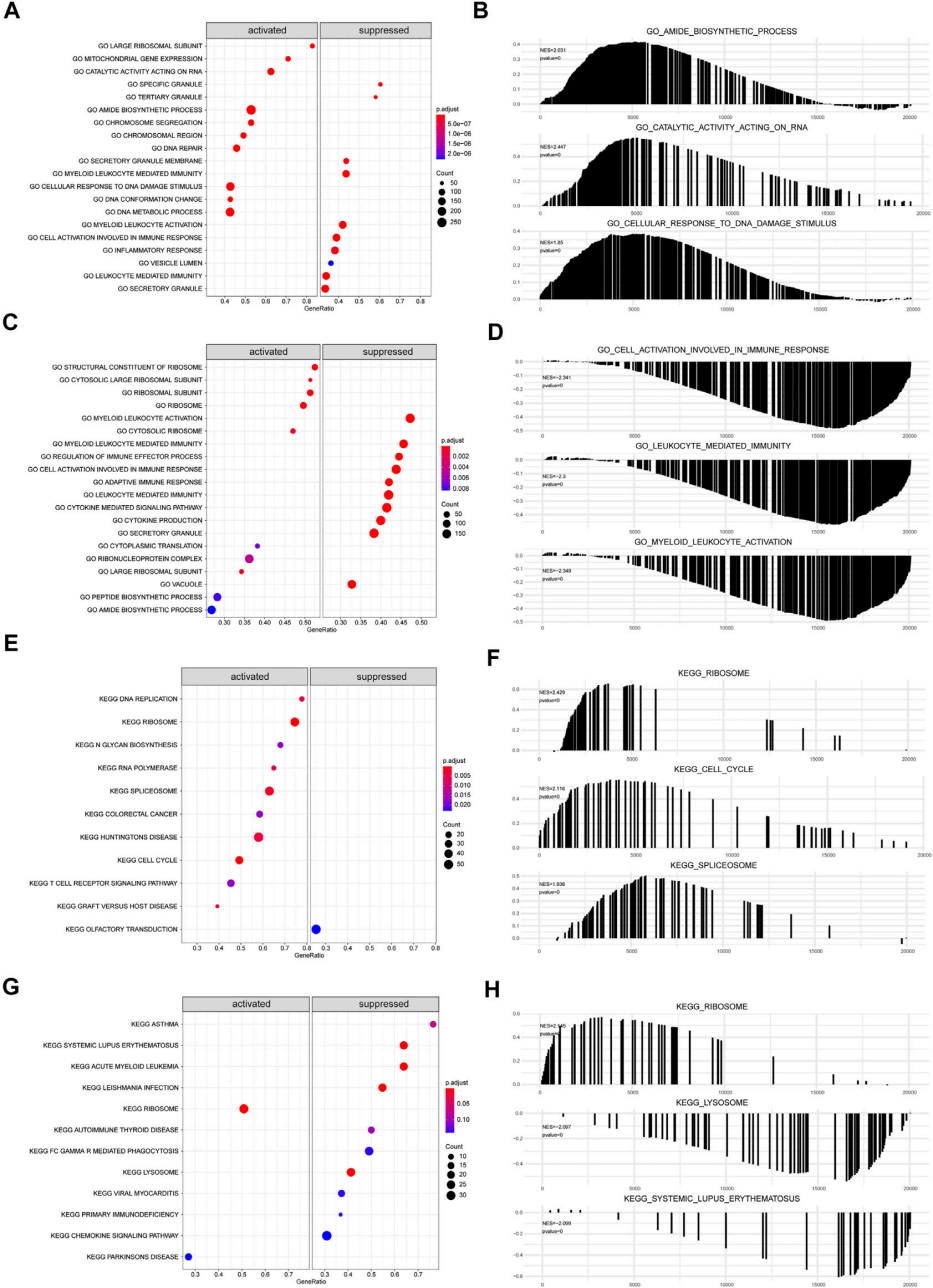
GSEA analysis. **(A,C)** For GSEA-GO enrichment analysis of two sets of mRNA data, the node color indicates the significant level of GO-term enrichment, and the node size indicates the number of genes enriched in the GO-term. **(B,D)** Top three presentations of GSEA-GO enrichment analysis results for both mRNA datasets. **(E,G)** GSEA-KEGG enrichment analysis of two sets of mRNA data. The node colors indicate the significance of pathway enrichment, while the node size indicates the number of genes enriched in the pathways. **(F,H)** Top three presentations of GSEA-KEGG enrichment analysis results for both mRNA datasets.

The biological functions and pathways that were highly altered in IDD samples compared to those observed in healthy samples from GSE124272 and GSE150408 were determined using GSEA (Figures 7A,C,E,G). The results indicated that the genes of GSE124272 were significantly involved in top three biological functions: amide biosynthetic process, catalytic activity acting on RNA, and cellular response to DNA damage stimulus (Figure 7B) and top three biological pathways: ribosome, cell cycle, and spliceosome (Figure 7F). The results indicated that the genes of GSE150408 were significantly involved in top three biological functions: cell activation involved in the immune response, leukocyte-mediated immunity, and myeloid leukocyte activation (Figure 7D) and top three biological pathways: ribosome, lysosome, and systemic lupus erythematosus (Figure 7H). The GSEA enrichment analysis is shown in Tables 5, 6.

**TABLE 5:** GSEA enrichment analysis of the GSE124272 dataset.

Description	ES	*p*-value
GO_AMIDE_BIOSYNTHETIC_PROCESS	2.030857969	1E-10
GO_CATALYTIC_ACTIVITY_ACTING_ON_RNA	2.446784621	1E-10
GO_CELLULAR_RESPONSE_TO_DNA_DAMAGE_STIMULUS	1.849853651	1E-10
GO_CELL_ACTIVATION_INVOLVED_IN_IMMUNE_RESPONSE	−1.9519684	1E-10
GO_CHROMOSOMAL_REGION	2.253151497	1E-10
GO_CHROMOSOME_SEGREGATION	2.210135641	1E-10
GO_DNA_CONFORMATION_CHANGE	2.43225748	1E-10
GO_DNA_METABOLIC_PROCESS	1.943934265	1E-10
GO_DNA_REPAIR	2.096888391	1E-10
GO_LARGE_RIBOSOMAL_SUBUNIT	2.594688023	1E-10
GO_LEUKOCYTE_MEDIATED_IMMUNITY	−1.991205529	1E-10
GO_MITOCHONDRIAL_GENE_EXPRESSION	2.524197795	1E-10
GO_MITOCHONDRIAL_MATRIX	2.388576169	1E-10
GO_MITOCHONDRIAL_PROTEIN_COMPLEX	2.405306648	1E-10
GO_MITOCHONDRIAL_TRANSLATION	2.51663285	1E-10
GO_MRNA_METABOLIC_PROCESS	1.988760775	1E-10
GO_MRNA_PROCESSING	1.981480179	1E-10
GO_MYELOID_LEUKOCYTE_ACTIVATION	−2.17430493	1E-10
GO_MYELOID_LEUKOCYTE_MEDIATED_IMMUNITY	−2.2767030	1E-10
GO_NCRNA_METABOLIC_PROCESS	2.585651374	1E-10
GO_NCRNA_PROCESSING	2.562235927	1E-10
GO_PEPTIDE_BIOSYNTHETIC_PROCESS	2.0977908	1E-10
GO_PEPTIDE_METABOLIC_PROCESS	1.816352777	1E-10
KEGG_RIBOSOME	2.428880984	3.206E-09
KEGG_CELL_CYCLE	2.116425583	1.162E-06
KEGG_SPLICEOSOME	1.93626882	5.034E-05
KEGG_HUNTINGTONS_DISEASE	1.806518486	0.0001529
KEGG_GRAFT_VERSUS_HOST_DISEASE	1.997480292	0.0001760
KEGG_DNA_REPLICATION	1.999216337	0.0002481
KEGG_T_CELL_RECEPTOR_SIGNALING_PATHWAY	1.737232303	0.0007438
KEGG_RNA_POLYMERASE	1.946578722	0.0008044
KEGG_COLORECTAL_CANCER	1.817577618	0.0009745
KEGG_N_GLYCAN_BIOSYNTHESIS	1.846326564	0.0012696

**TABLE 6:** GSEA enrichment analysis of the GSE150408 dataset.

Description	ES	*p-*value
GO_CELL_ACTIVATION_INVOLVED_IN_IMMUNE_RESPONSE	−2.3414237	1E-10
GO_LEUKOCYTE_MEDIATED_IMMUNITY	−2.3003049	1E-10
GO_MYELOID_LEUKOCYTE_ACTIVATION	−2.3492811	1E-10
GO_MYELOID_LEUKOCYTE_MEDIATED_IMMUNITY	−2.4082594	1E-10
GO_SECRETORY_GRANULE	−2.0382624	1E-10
GO_REGULATION_OF_IMMUNE_EFFECTOR_PROCESS	−2.111030	3.219E-10
GO_ADAPTIVE_IMMUNE_RESPONSE	−2.0372048	7.394E-10
GO_CYTOKINE_MEDIATED_SIGNALING_PATHWAY	−1.8231034	1.263E-09
GO_CYTOKINE_PRODUCTION	−1.8338217	1.523E-09
GO_VACUOLE	−1.8045488	2.143E-09
GO_VESICLE_MEMBRANE	−1.7610719	4.196E-09
GO_POSITIVE_REGULATION_OF_IMMUNE_RESPONSE	−1.8157690	5.302E-09
GO_VESICLE_LUMEN	−1.9977589	2.817E-08
GO_SMALL_GTPASE_BINDING	−1.9056700	6.658E-08
GO_ADAPTIVE_IMMUNE_RESPONSE_BASED_ON_SOMATIC_RECOMBINATION_OF_IMMUNE_RECEPTORS_BUILT_FROM_IMMUNOGLOBULIN_SUPERFAMILY_DOMAINS	−2.0518571	7.30E-08
GO_INFLAMMATORY_RESPONSE	−1.7399725	9.696E-08
GO_REGULATION_OF_CELL_ADHESION	−1.7087391	3.127E-07
GO_RIBOSOME	1.959447	3.444E-07
GO_LEUKOCYTE_DIFFERENTIATION	−1.7751505	4.401E-07
GO_SPECIFIC_GRANULE	−2.0905806	5.423E-07
GO_TERTIARY_GRANULE_LUMEN	−2.278010	5.610E-07
GO_GTPASE_BINDING	−1.74771547	6.768E-07
GO_REGULATION_OF_HEMOPOIESIS	−1.8218795	7.080E-07
KEGG_RIBOSOME	2.1454455	1.907E-06
KEGG_LYSOSOME	−2.0967835	2.7265E-06
KEGG_SYSTEMIC_LUPUS_ERYTHEMATOSUS	−2.0991050	5.188E-05
KEGG_LEISHMANIA_INFECTION	−2.0482099	5.82E-05
KEGG_ACUTE_MYELOID_LEUKEMIA	−2.0433323	0.0001107
KEGG_ASTHMA	−1.8595101	0.00211956
KEGG_FC_GAMMA_R_MEDIATED_PHAGOCYTOSIS	−1.6496656	0.0042681
KEGG_PARKINSONS_DISEASE	1.5796279	0.0067326
KEGG_VIRAL_MYOCARDITIS	−1.6506216	0.0100046
KEGG_PRIMARY_IMMUNODEFICIENCY	−1.7349073	0.0112876

### GSVA Analysis

**FIGURE 8:**
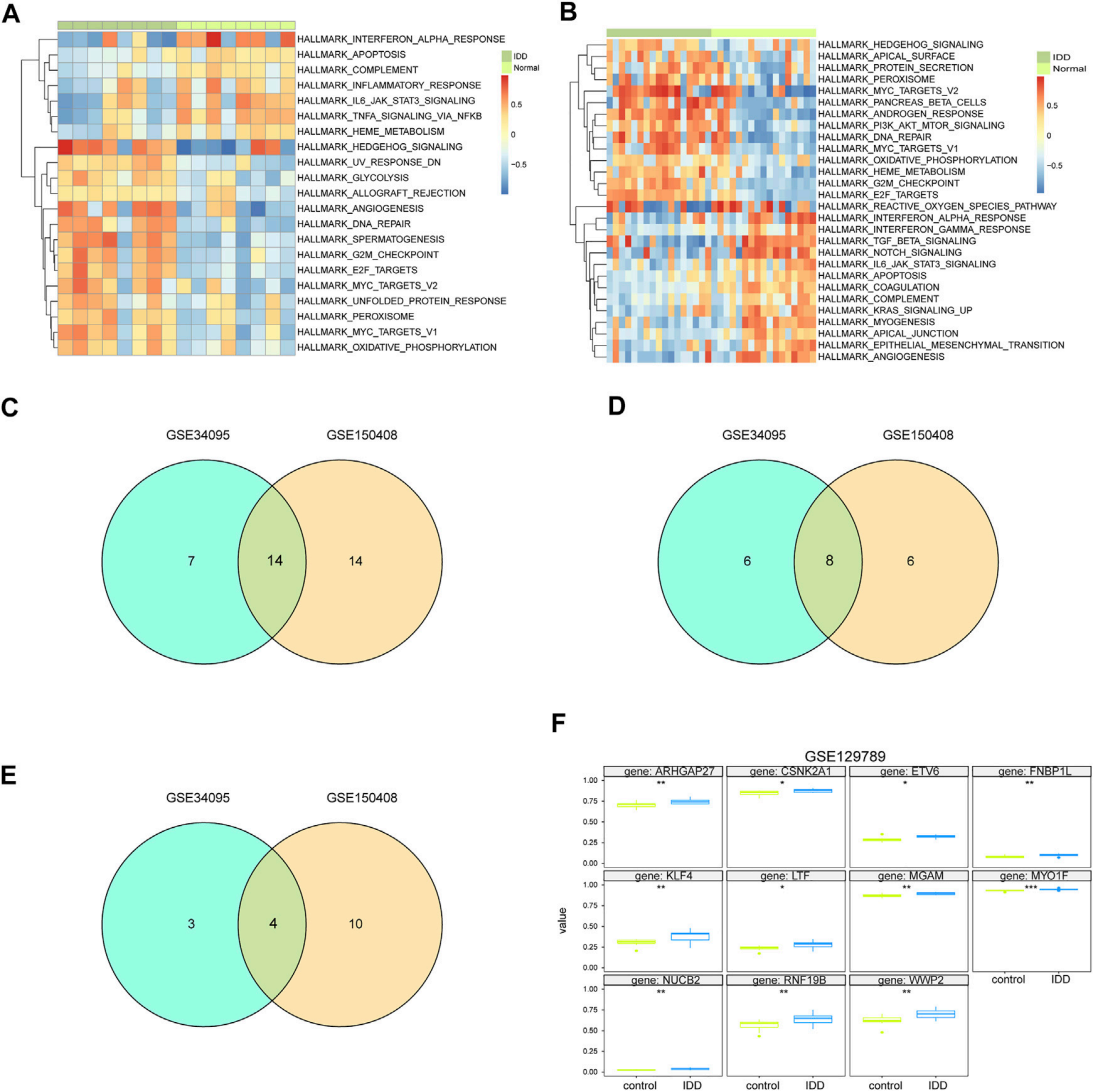
GSVA analysis of hub genes. **(A)** GSVA analysis of mRNA dataset GSE124272 indicates 50 hallmark gene sets. **(B)** GSVA analysis of mRNA dataset GSE150408 indicates 50 hallmark gene sets. **(C)** Venn diagram of hallmark for significant differences in normal disc tissue and disc-degenerated tissue from GSE124272 and GSE150408 GSVA analyses. **(D)** Venn diagram of hallmark with a lower enrichment score in normal intervertebral disc tissue than that in IDD tissue from GSE124272 and GSE150408 datasets. **(E)** Venn diagram of hallmark with higher enrichment scores in normal intervertebral disc tissue than that in IDD tissue from GSE124272 and GSE150408 datasets. **(F)** Methylation level analysis of hub genes. Hub genes whose methylation levels were significantly different in normal intervertebral disc tissue and IDD tissue are shown.

We carried out GSVA of IDD samples and compared it with healthy samples from GSE124272 and GSE1150408. The results were displayed as a heatmap (Figures 8A,B). All IDD samples were separated into low- and high-score groups according to the median GSVA score. In total, we obtained 28 high-score hallmark gene sets and 21 low-score gene sets (Figure 8C), of which 8 hallmark gene sets scored high in both datasets and 4 hallmark gene sets scored low in both datasets (Figures 8D,E). The 8 high-scoring hallmark gene sets included DNA repair, hedgehog signaling, E2F targets, myc targets, oxidative phosphorylation, and peroxisome. The 4 low-scoring hallmark gene sets included IL-6-JAK-STAT3 signaling, apoptosis, interferon-α response, and complement system.

### Methylation Analysis of Hub Genes

The aligned methylation data of the 71 hub genes in IDD were obtained from GSE129789, and our analysis indicated significant hypermethylation of ARHGAP27, CSNK2A1, ETV6, FNBP1L, KLF4, LTF, MGAM, MYO1F, NUCB2, RNF19B, and WWP3 (Figure 8F). The methylation of hub genes may be an important biomarker and potential therapeutic target for IDD.

### ROC Analysis and Friend Analysis of Hub Genes

**FIGURE 9:**
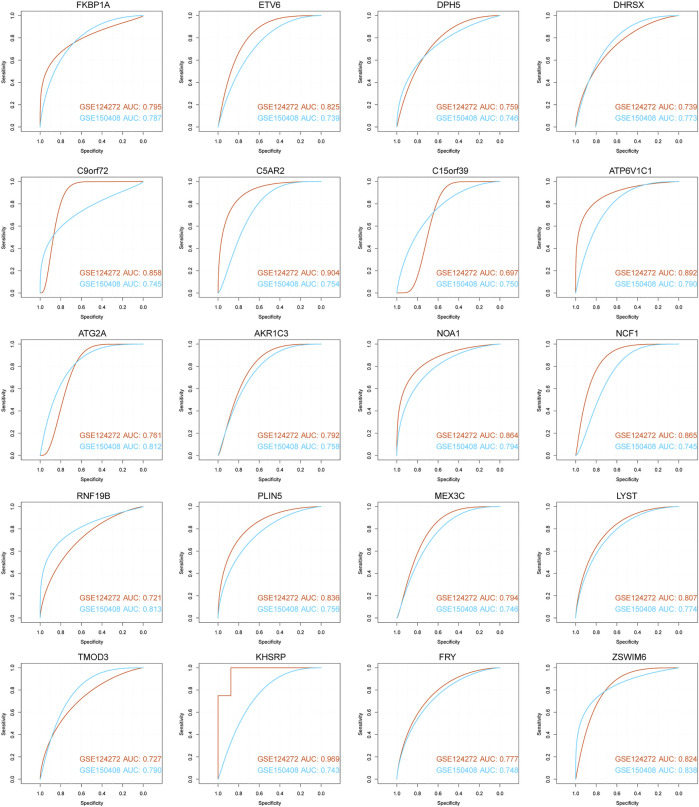
ROC curve for diagnosis of 20 hub genes. The horizontal axis represents the test’s specificity, and the vertical axis represents the test’s sensitivity. The brick red curve is the test result for data 1, and the blue curve is the test result for data 2.

**FIGURE 10:**
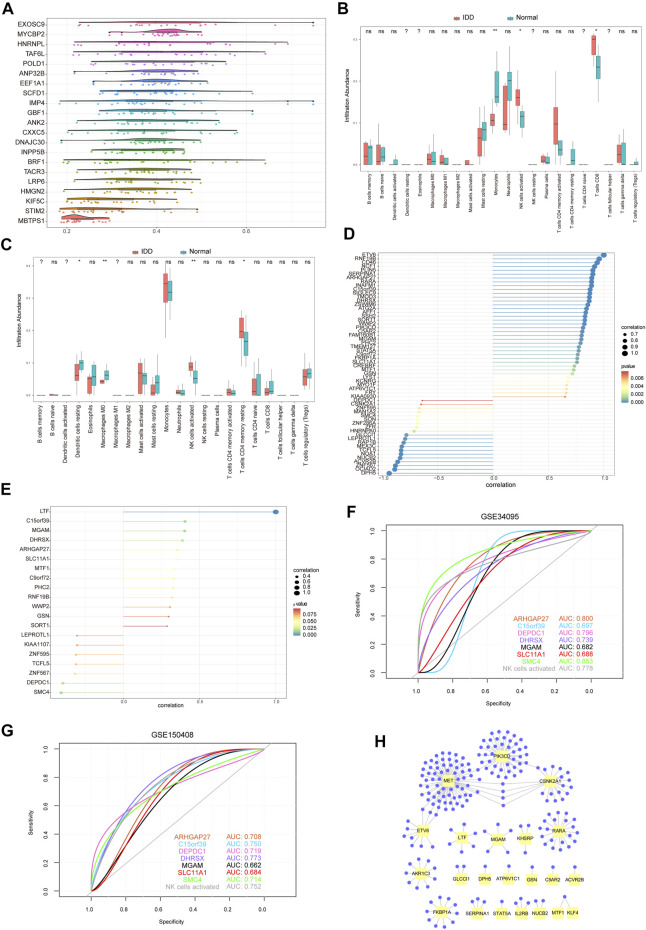
Immune infiltration analysis of hub genes. **(A)** Friend analysis of 19 hub genes. The horizontal axis represents the correlation strength, and the vertical axis indicates the gene name. **(B)** Immune infiltration analysis of 22 immune cells by 71 hub genes from the GSE124272 dataset. Pink indicates degenerative tissue of intervertebral disc, and blue–green indicates normal tissue. **(C)** Immune infiltration analysis of 71 hub genes into 22 immune cells using the GSE150408 dataset. The pink color indicates IDD tissue, and the blue–green color indicates normal tissue. **(D)** Pearson correlation coefficient between active NK cell enrichment abundance and 58 hub gene expression values related to IDD. GSE124272 dataset, in which the node size indicates the absolute value of correlation and the node color indicates the significant correlation. **(E)** Pearson correlation coefficient between the abundance of active NK cell enrichment and the expression values of 20 hub genes related to IDD. GSE150408 dataset, in which the node size indicates the absolute value of correlation and the node color indicates the significant correlation. **(F)** ROC curve of the seven hub genes and NK cell activation (from GSE124272 dataset). Test specificity and sensitivity are presented on the horizontal and vertical axes, respectively. **(G)** ROC curve of the seven hub genes and NK cell activation (from the GSE150408 dataset). Test specificity and sensitivity are presented on the horizontal and vertical axes, respectively. **(H)** Hub gene–potential drug network; the yellow node represents the hub gene, and the violet node represents the potential drug.

We used ROC curve analysis to assess the diagnostic accuracy of the 71 hub genes. The results show that the AUC of 20 genes (AKR1C3, ATG2A, ATP6V1C1, C15orf39, C5AR2, C9orf72, DHRSX, DPH5, ETV6, FKBP1A, FRY, KHSRP, MEX3C, NCF1, LYST, NOA1, PLIN5, RNF19B, TMOD3, and ZSWIM6) in both datasets was greater than 0.5, showing good classification efficiency (Figure 9). Next, we analyzed the functional similarity of the aforementioned genes. The results showed that 20 genes had low functional similarity and were highly representative (Figure 10A).

### Immune Cell Infiltration Analysis

To examine the relationship between 71 hub genes and 22 immune cell types that frequently infiltrate the IDD microenvironment, we first performed immune cell infiltration analyses on GSE124272 and GSE150408 datasets (Figures 10B,C). We found significantly different concentrations of NK cells activated between healthy and IDD samples in both datasets. Next, we analyzed the relationship between 71 hub genes on GSE124272 and GSE150408 datasets and NK cell activation using the Pearson correlation analysis (Figures 10D,E) and obtained 7 hub genes (ARHGAP27, C15orf39, DEPDC1, DHRSX, MGAM, SLC11A1, and SMC4) that were closely related to NK cell activation in both datasets. We found that the aforementioned 7 hub genes associated with NK cell activation distinguished the degenerative intervertebral disc tissue from healthy tissue data from the GSE124272 and GSE150408 datasets using the ROC analysis (Figures 10F,G).

### Identification of the Potential Drugs

We used the DGIdb database to determine the potential drug or molecular compounds that could regulate the DEGs. The results displayed a drug–hub gene interaction network (Figure 10H). We identified 205 potential drugs or molecular compounds corresponding to 24 mRNAs. Among them, 10 drugs or molecular compounds targeted two key genes at the same time, including ALTIRATINIB, APTO-253, CRIZOTINIB, CYC-116, DACTOLISIB, ENTRECTINIB, PF-00562271, QUERCETIN, R-406, and SP-600125. In addition, we found that 73 drugs or molecular compounds targeted the MET gene and 73 drugs or molecular compounds targeted the PIK3CD gene.

### Expression Level of Key DEGs

**FIGURE 11:**
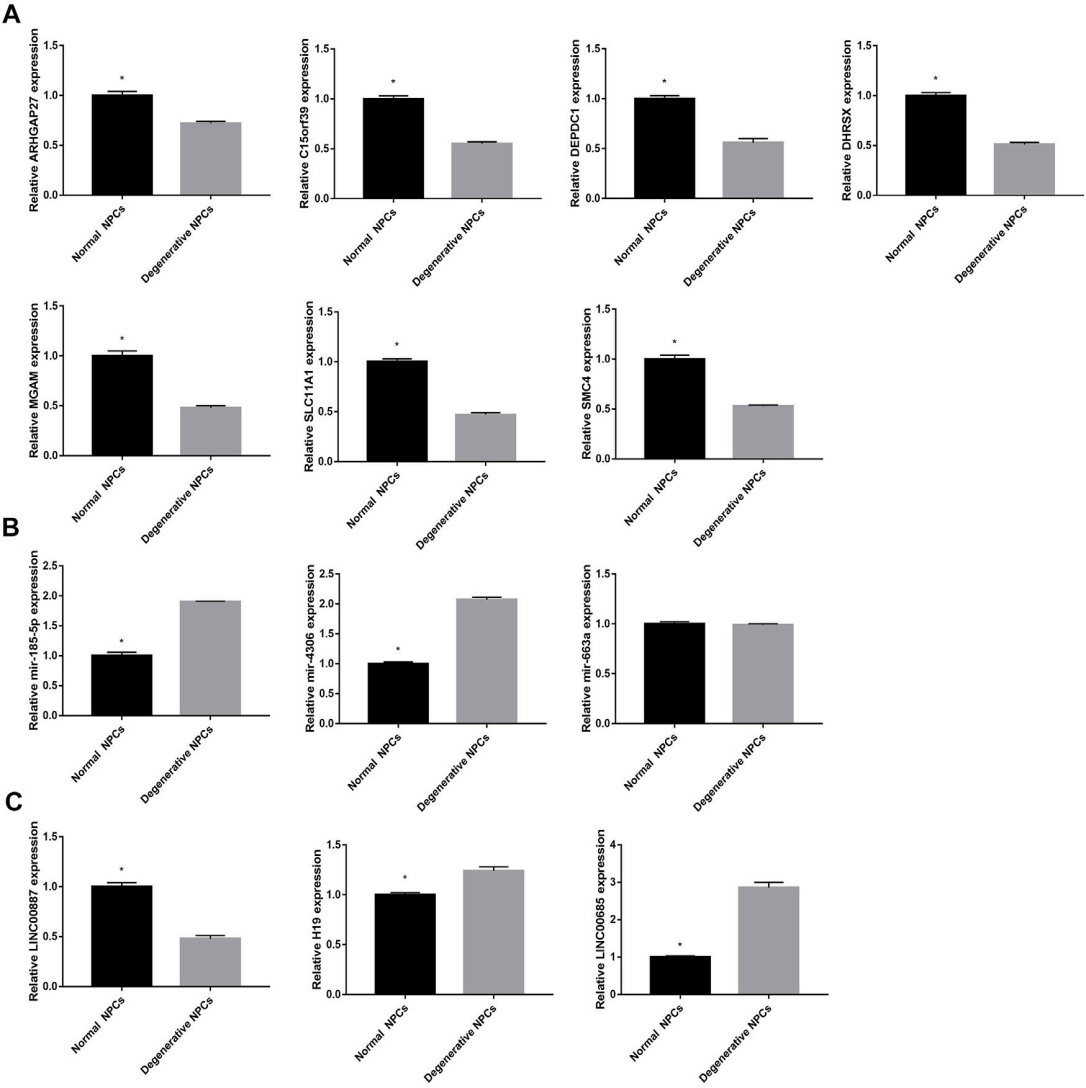
Expression level of hub DEGs in normal and degeneration NPCs. **(A)** Expression level of mRNAs in normal and degeneration NPCs. **(B)** Expression level of miRNAs in normal and degeneration NPCs. **(C)** Expression level of lncRNAs in normal and degeneration NPCs. *p < 0.05.

In order to further verify the accuracy of the aforementioned results, we detected the expression of 13 DEGs by qRT-PCR. The results showed that 7 DEGs closely related to NK cell activation were downregulated in degenerative NPCs. In addition, the results showed that LINC00887 was significantly downregulated in degenerative NPCs; H19, LINC00685, mir-185-5p, and mir-4306 were upregulated in degenerative NPCs; and the expression level of mir-663a did not change significantly in normal and degenerative NPCs (Figure 11).

## Discussion

IDD is a high-incidence degenerative disease, often resulting in low back pain and even disability. However, in most cases, the clinical results are suboptimal. Therefore, understanding the pathological changes and molecular mechanisms of IDD is crucial for clinical diagnosis and treatment. Efficient microarray and bioinformatics analyses help us understand the molecular mechanisms of the disease onset and development, which is invaluable for biomarkers and therapeutic target identification.

First, we identified DEGs associated with IDD by analyzing seven expression profiles and constructed a PPI network and performed GO and KEGG enrichment analyses of the DEGs from the PPI network. Previous studies have proved that alternatives in national NP cell behavior are related to inflammation and immune response, while IL-1, macrophages, and lymphocytes have been verified to be upregulated in IDD (Phillips et al., 2013). The top three genes in the PPI network were TLR4, STAT3, and TLR8. Also, previous studies have shown that the inflammatory response (Bin et al., 2021), immune response (Sun et al., 2020), and IL-6/JAK/STAT3 pathway are related to the degeneration of human NP cells (Wu et al., 2021). In addition, a study of single-cell RNA sequencing of IDD pointed out that chondrocytes in one of the differentiated orientations interact with macrophages and endothelial cells and have an inflammatory amplification effect (Han et al., 2022). Taken together, it is speculated that the IL and TLR families may play a significant role in the development of IDD.

Next, we constructed the lncRNA–miRNA–mRNA network. Also, 11 widely regulated RNAs were screened. Of these, miR-185-5 P and NEAT1 have been shown to be associated with IDD progression (Zhang H. et al., 2020; Li et al., 2021). The remaining genes have been shown to be involved in the pathological process of other diseases (Ren et al., 2019; Tollenaere et al., 2019; Yang et al., 2019a; Zhang et al., 2019). Currently, it has not been proven that these genes are related to IDD. However, inflammatory responses, macrophage activation, the AKT/NF-kB signaling pathway, collagen II, and aggrecans have been shown to be associated with the progress of IDD. Therefore, we speculate that the aforementioned genes may represent potential biomarkers for IDD. We then selected 71 differentially co-expressed genes as hub genes and performed GO, KEGG, GSEA, GSVA, and DO on the hub genes. The changes of hub genes were significantly enriched in cytoplasmic vesicles, lipid particles, and the plasma membrane. Since lipopolysaccharide-induced NP cell pyroptosis could be inhibited by MSC-derived exosomes *in vitro* (Zhang J. et al., 2020), we speculate that exosomes may represent a new therapeutic site for IDD. The methylation analysis of the hub genes showed that 11 genes (ARHGAP27, CSNK2A1, ETV6, FNBP1L, KLF4, LTF, MGAM, MYO1F, NUCB2, RNF19B, and WWP3) were hypermethylated in IDD. Nevertheless, there is no clear proof that these genes are related to IDD. Our data mining further shows that transcription factors and DNA binding play a significant role in IDD. The 11 key genes have been confirmed to be related to inflammatory reactions, autophagy, and macrophages (Huett et al., 2009; Lawrence et al., 2020; Sahoo et al., 2020; Taracha-Wisniewska et al., 2020). Therefore, we infer that they most likely play a role in IDD by regulating inflammatory responses, autophagy, and numerous other aspects. We believe this will provide a reference for understanding the mechanism underlying IDD development and guiding the treatment of IDD.

Among the five KEGG pathways, the chemokine pathway was identified as being strongly associated with the development of IDD. Previous studies have confirmed that multiple molecules involved in these pathways were associated with pathological changes in that intervertebral disc tissue (Gao et al., 2016; Wang J. et al., 2019; Liu et al., 2020). Our data mining results further confirm that the chemokine pathway plays a crucial role in the development of IDD.

The results of the GSEA highlighted the important roles of inflammatory and immune responses in IDD. GSVA suggested that the gene sets highly correlated with IDD were associated with DNA repair, cell cycle, oxidative phosphorylation, IL-6-JAK-STAT3 signaling, and apoptosis. Among them, DNA repair and JAK-STAT3 signaling have been proved to be highly correlated with IDD (Nasto et al., 2013; Dai et al., 2021). Mitochondria are the principal source of energy and ROS production in IVD cells. Mitochondrial dysfunction leads to oxidative stress, cell death, and premature aging (Saberi et al., 2021). In IDD, mitotic damage leads to progressive aggregation of defective mitochondria, NP cell apoptosis, ECM degradation, and cartilage degradation (Sun et al., 2021). Moving forward, we can start from these related genes in our search for new treatments for IDD. Among the 71 hub genes identified in this study, PLIN5 has been shown to boost the transcription of genes involved in mitochondrial biogenesis and oxidative metabolism (Gallardo-Montejano et al., 2021). Furthermore, NOA1 was shown to be necessary for mitochondrial basic functions such as ATP synthesis and apoptosis (Kolanczyk et al., 2011). PLIN5 and NOA1 demonstrated good diagnostic efficacy in the ROC analysis. Therefore, we speculate that they might represent therapeutic targets for IDD.

Following immune cell infiltration analysis, we obtained seven overlapping genes (ARHGAP27, C15orf39, DEPDC1, DHRSX, MGAM, SLC11A1, and SMC4) that were strongly associated with active NK cells in both datasets. Among these, the expression of SLC11A1 protein was identified in NK and NK-like cells, and the expression of SLC11A1 might be closely related to the enhanced activation of congenital lymphocytes (Hedges et al., 2013). Although there is no clear evidence that SLC11A1 is involved in the development of IDD, the authors believed that SLC11A1 has a research value. Next, we explored the functions of the hub genes and found that numerous genes were associated with the autophagy process including DEPDC, DHRSX, ATG2A, and C9orf72. These four genes have been reported to be involved in the regulation of autophagy (Zhang et al., 2014; Bakula et al., 2017; Wang W. et al., 2020; Pang and Hu, 2021), and they also showed good classification efficiency in ROC analysis. In addition, DEPDC and DHRSX were highly correlated with NK cell activation. Previous studies have confirmed that autophagy plays a significant role in the development of IDD (Tang et al., 2019); therefore, we pointed out that experiments should further investigate the roles of these four genes in IDD.

Another interesting phenomenon was that MTF-1, MGAM, NUCB2, and TMOD3 were discovered to be related to the body glucose regulation (Xu et al., 2019; Yang et al., 2019b; Shrestha et al., 2021). Multiple studies have shown that diabetes is an important risk factor for IVDD (Cannata et al., 2020; Shao et al., 2021). Of interest, DO analysis of IDD samples showed enrichment for myeloproliferative disorders, acute leukemia, salivary gland malignancies, *E. coli* infection, immunodeficiency syndrome, subretinal membrane, chronic myeloproliferative disorders, malignant mesothelioma, polycythemia vera, familial dementia, and others. These conditions have not been previously shown to be high-risk factors for IDD. We wonder whether these conditions contribute to the progression of IDD in the same way diabetes does. If so, how can the treatment strategy be adjusted when treating patients who have such diseases comorbid with IDD?

The top two targeted hub genes for potential drug or molecular compounds were MET and PIK3CD, both regulated by 73 potential drugs or molecular compounds. HGF/c-Met signaling may utilize the HIF-1α expression to regulate cell proliferation in the intervertebral disc (Tonomura et al., 2020). The PIK3CD gene encodes a p110δ catalytic subtype of PI3Ks. P110δ is mainly enriched in leukocytes and regulates immune function. Also, the PI3K family has been confirmed to be involved in the progression of IDD (Ouyang et al., 2017). However, the specific mechanisms via which MET and PIK3CD act in IDD required further experimental investigation. We speculated that once the specific mechanisms of MET and PIK3CD in IDD are elucidated, these 73 drugs and molecules are likely to represent new options for the treatment of IDD.

This study had several limitations. First, the data used were downloaded from a public database, and it was not possible to assess whether there were entry errors. Also, there exist differences in the enriched gene functions and pathways of the differentially expressed genes between mRNA and protein data, and this study focused only on mRNA datasets (Xu et al., 2021). Second, our study only focused on the genes that were identified as differentially expressed in datasets. Thus, some characteristics such as sex, age, and degree of IDD have not been taken into consideration in our study. Third, more relevant experimental evidence was needed to fully clarify the role of central genes and the potential mechanism of IDD. Finally, to avoid analysis bias led by the retrospective nature of the current study, we should perform a prospective study in the future.

In conclusion, the molecular mechanisms underlying IDD have been explored in the present study using comprehensive bioinformatics analysis to clarify the related biological functions and pathways associated with the occurrence and development of IDD. By analyzing seven gene expression datasets, we studied the differential expression of mRNAs, miRNAs, and lncRNA related to the progression of IDD and constructed a PPI network as well as a lncRNA–miRNA–mRNA network and revealed the possible functions and pathways associated with the disease. Subsequently, we identified 71 hub genes that might play important roles in the disease onset and development. Next, we performed methylation, immune infiltration, and ROC analyses and predicted potential therapeutic drugs. A comprehensive analysis was performed to explore key potential mechanisms possibly involved in the occurrence and progression of IDD through comprehensive analysis. Further studies are urgently needed to verify and reveal further mechanisms. Our results may help identify new potential therapeutic targets for IDD.

## Data Availability

The raw data supporting the conclusion of this article will be made available by the authors, without undue reservation.

## References

[B1] Arraiano C. M. (2021). Regulatory Noncoding RNAs: Functions and Applications in Health and Disease. FEBS J. 288, 6308–6309. 10.1111/febs.16027 34153158

[B2] Ashburner M. Ball C. A. Blake J. A. Botstein D. Butler H. Cherry J. M. (2000). Gene Ontology: Tool for the Unification of Biology. The Gene Ontology Consortium. Nat. Genet. 25, 25–29. 10.1038/75556 10802651 PMC3037419

[B3] Bakula D. Müller A. J. Zuleger T. Takacs Z. Franz-Wachtel M. Thost A.-K. (2017). WIPI3 and WIPI4 β-propellers Are Scaffolds for LKB1-AMPK-TSC Signalling Circuits in the Control of Autophagy. Nat. Commun. 8, 15637. 10.1038/ncomms15637 28561066 PMC5460038

[B4] Barrett T. Troup D. B. Wilhite S. E. Ledoux P. Rudnev D. Evangelista C. (2007). NCBI GEO: Mining Tens of Millions of Expression Profiles-Ddatabase and Tools Update. Nucleic Acids Res. 35, D760–D765. 10.1093/nar/gkl887 17099226 PMC1669752

[B5] Bin S. Xin L. Lin Z. Jinhua Z. Rui G. Xiang Z. (2021). Targeting miR-10a-5p/IL-6R axis for Reducing IL-6-Induced Cartilage Cell Ferroptosis. Exp. Mol. Pathology 118, 104570. 10.1016/j.yexmp.2020.104570 33166496

[B6] Bindea G. Mlecnik B. Hackl H. Charoentong P. Tosolini M. Kirilovsky A. (2009). ClueGO: A Cytoscape Plug-In to Decipher Functionally Grouped Gene Ontology and Pathway Annotation Networks. Bioinformatics 25, 1091–1093. 10.1093/bioinformatics/btp101 19237447 PMC2666812

[B7] Cannata F. Vadalà G. Ambrosio L. Fallucca S. Napoli N. Papalia R. (2020). Intervertebral Disc Degeneration: A Focus on Obesity and Type 2 Diabetes. Diabetes Metab. Res. Rev. 36, e3224. 10.1002/dmrr.3224 31646738

[B8] Chang H. Wang H. Yang X. You K. Jiang M. Cai F. (2021). Comprehensive Profile Analysis of Differentially Expressed circRNAs in Glucose Deprivation-Induced Human Nucleus Pulposus Cell Degeneration. Biomed. Res. Int. 2021, 4770792. 10.1155/2021/4770792 34285912 PMC8275381

[B9] Cotto K. C. Wagner A. H. Feng Y.-Y. Kiwala S. Coffman A. C. Spies G. (2018). DGIdb 3.0: A Redesign and Expansion of the Drug-Gene Interaction Database. Nucleic Acids Res. 46, D1068–D1073. 10.1093/nar/gkx1143 29156001 PMC5888642

[B10] Dai S. Liang T. Shi X. Luo Z. Yang H. (2021). Salvianolic Acid B Protects Intervertebral Discs from Oxidative Stress-Induced Degeneration via Activation of the JAK2/STAT3 Signaling Pathway. Oxid. Med. Cell. Longev. 2021, 6672978. 10.1155/2021/6672978 33628378 PMC7896869

[B11] Du X.-F. Cui H.-T. Pan H.-H. Long J. Cui H.-W. Chen S.-L. (2021). Role of the miR-133a-5p/FBXO6 Axis in the Regulation of Intervertebral Disc Degeneration. J. Orthop. Transl. 29, 123–133. 10.1016/j.jot.2021.05.004 PMC8233105 34249610

[B12] Fan Y. Xia J. (2018). miRNet-Functional Analysis and Visual Exploration of miRNA-Target Interactions in a Network Context. Methods Mol. Biol. 1819, 215–233. 10.1007/978-1-4939-8618-7_10 30421406

[B13] Gallardo-Montejano V. I. Yang C. Hahner L. Mcafee J. L. Johnson J. A. Holland W. L. (2021). Perilipin 5 Links Mitochondrial Uncoupled Respiration in Brown Fat to Healthy White Fat Remodeling and Systemic Glucose Tolerance. Nat. Commun. 12, 3320. 10.1038/s41467-021-23601-2 34083525 PMC8175597

[B14] Gao G. He J. Nong L. Xie H. Huang Y. Xu N. (2016). Periodic Mechanical Stress Induces the Extracellular Matrix Expression and Migration of Rat Nucleus Pulposus Cells by Upregulating the Expression of Intergrin α1 and Phosphorylation of Downstream Phospholipase Cγ1. Mol. Med. Rep. 14, 2457–2464. 10.3892/mmr.2016.5549 27484337 PMC4991676

[B15] Ge J. Cheng X. Yan Q. Wu C. Wang Y. Yu H. (2020). Calcitonin Inhibits Intervertebral Disc Degeneration by Regulating Protein Kinase C. J. Cell. Mol. Med. 24, 8650–8661. 10.1111/jcmm.15496 32564456 PMC7412402

[B16] Han S. Zhang Y. Zhang X. Zhang H. Meng S. Kong M. (2022). Single-Cell RNA Sequencing of the Nucleus Pulposus Reveals Chondrocyte Differentiation and Regulation in Intervertebral Disc Degeneration. Front. Cell. Dev. Biol. 10, 824771. 10.3389/fcell.2022.824771 35265617 PMC8899542

[B17] Hedges J. F. Kimmel E. Snyder D. T. Jerome M. Jutila M. A. (2013). Solute Carrier 11A1 Is Expressed by Innate Lymphocytes and Augments Their Activation. J. Immunol. 190, 4263–4273. 10.4049/jimmunol.1200732 23509347 PMC3622125

[B18] Huett A. Ng A. Cao Z. Kuballa P. Komatsu M. Daly M. J. (2009). A Novel Hybrid Yeast-Human Network Analysis Reveals an Essential Role for FNBP1L in Antibacterial Autophagy. J. Immunol. 182, 4917–4930. 10.4049/jimmunol.0803050 19342671 PMC2752416

[B19] Ji M.-l. Jiang H. Zhang X.-j. Shi P.-l. Li C. Wu H. (2018). Preclinical Development of a microRNA-Based Therapy for Intervertebral Disc Degeneration. Nat. Commun. 9, 5051. 10.1038/s41467-018-07360-1 30487517 PMC6262020

[B20] Kanehisa M. Goto S. (2000). KEGG: Kyoto Encyclopedia of Genes and Genomes. Nucleic Acids Res. 28, 27–30. 10.1093/nar/28.1.27 10592173 PMC102409

[B21] Kolanczyk M. Pech M. Zemojtel T. Yamamoto H. Mikula I. Calvaruso M.-A. (2011). NOA1 Is an Essential GTPase Required for Mitochondrial Protein Synthesis. Mol. Biol. Cell. 22, 1–11. 10.1091/mbc.e10-07-0643 21118999 PMC3016967

[B22] Lawrence D. W. Willard P. A. Cochran A. M. Matchett E. C. Kornbluth J. (2020). Natural Killer Lytic-Associated Molecule (NKLAM): An E3 Ubiquitin Ligase with an Integral Role in Innate Immunity. Front. Physiol. 11, 573372. 10.3389/fphys.2020.573372 33192571 PMC7658342

[B23] Li C. Ma X. Ni C. Xu J. Xie Y. Kan J. (2021). LncRNA NEAT1 Promotes Nucleus Pulposus Cell Matrix Degradation through Regulating Nrf2/ARE axis. Eur. J. Med. Res. 26, 11. 10.1186/s40001-021-00481-2 33478594 PMC7818737

[B24] Liberzon A. Birger C. Thorvaldsdóttir H. Ghandi M. Mesirov J. P. Tamayo P. (2015). The Molecular Signatures Database (MSigDB) Hallmark Gene Set Collection. Cell. Syst. 1, 417–425. 10.1016/j.cels.2015.12.004 26771021 PMC4707969

[B25] Liu L. He J. Liu C. Yang M. Fu J. Yi J. (2021). Cartilage Intermediate Layer Protein Affects the Progression of Intervertebral Disc Degeneration by Regulating the Extracellular Microenvironment (Review). Int. J. Mol. Med. 47, 475–484. 10.3892/ijmm.2020.4832 33416131 PMC7797476

[B26] Liu W. Niu F. Sha H. Liu L. D. Lv Z. S. Gong W. Q. (2020). Apelin-13/APJ System Delays Intervertebral Disc Degeneration by Activating the PI3K/AKT Signaling Pathway. Eur. Rev. Med. Pharmacol. Sci. 24, 2820–2828. 10.26355/eurrev_202003_20643 32271399

[B27] Liu X. Che L. Xie Y.-K. Hu Q.-J. Ma C.-J. Pei Y.-J. (2015). Noncoding RNAs in Human Intervertebral Disc Degeneration: An Integrated Microarray Study. Genomics Data 5, 80–81. 10.1016/j.gdata.2015.05.027 26484230 PMC4583642

[B28] Lu T. X. Rothenberg M. E. (2018). MicroRNA. J. Allergy Clin. Immunol. 141, 1202–1207. 10.1016/j.jaci.2017.08.034 29074454 PMC5889965

[B29] Nasto L. A. Wang D. Robinson A. R. Clauson C. L. Ngo K. Dong Q. (2013). Genotoxic Stress Accelerates Age-Associated Degenerative Changes in Intervertebral Discs. Mech. Ageing Dev. 134, 35–42. 10.1016/j.mad.2012.11.002 23262094 PMC3558562

[B30] Newman A. M. Steen C. B. Liu C. L. Gentles A. J. Chaudhuri A. A. Scherer F. (2019). Determining Cell Type Abundance and Expression from Bulk Tissues with Digital Cytometry. Nat. Biotechnol. 37, 773–782. 10.1038/s41587-019-0114-2 31061481 PMC6610714

[B31] Ouyang Z.-H. Wang W.-J. Yan Y.-G. Wang B. Lv G.-H. (2017). The PI3K/Akt Pathway: A Critical Player in Intervertebral Disc Degeneration. Oncotarget 8, 57870–57881. 10.18632/oncotarget.18628 28915718 PMC5593690

[B32] Pang W. Hu F. (2021). Cellular and Physiological Functions of C9ORF72 and Implications for ALS/FTD. J. Neurochem. 157, 334–350. 10.1111/jnc.15255 33259633 PMC8842544

[B33] Phillips K. L. E. Chiverton N. Michael A. L. Cole A. A. Breakwell L. M. Haddock G. (2013). The Cytokine and Chemokine Expression Profile of Nucleus Pulposus Cells: Implications for Degeneration and Regeneration of the Intervertebral Disc. Arthritis Res. Ther. 15, R213. 10.1186/ar4408 24325988 PMC3979161

[B34] Ren H. Yu X. Shen G. Zhang Z. Shang Q. Zhao W. (2019). miRNA-Seq Analysis of Human Vertebrae Provides Insight into the Mechanism Underlying GIOP. Bone 120, 371–386. 10.1016/j.bone.2018.11.013 30503955

[B35] Ritchie M. E. Phipson B. Wu D. Hu Y. Law C. W. Shi W. (2015). Limma Powers Differential Expression Analyses for RNA-Sequencing and Microarray Studies. Nucleic Acids Res. 43, e47. 10.1093/nar/gkv007 25605792 PMC4402510

[B36] Robin X. Turck N. Hainard A. Tiberti N. Lisacek F. Sanchez J.-C. (2011). pROC: An Open-Source Package for R and S+ to Analyze and Compare ROC Curves. BMC Bioinforma. 12, 77. 10.1186/1471-2105-12-77 PMC3068975 21414208

[B37] Saberi M. Zhang X. Mobasheri A. (2021). Targeting Mitochondrial Dysfunction with Small Molecules in Intervertebral Disc Aging and Degeneration. Geroscience 43, 517–537. 10.1007/s11357-021-00341-1 33634362 PMC8110620

[B38] Sahoo P. K. Kar A. N. Samra N. Terenzio M. Patel P. Lee S. J. (2020). A Ca(2+)-Dependent Switch Activates Axonal Casein Kinase 2alpha Translation and Drives G3BP1 Granule Disassembly for Axon Regeneration. Curr. Biol. 30, 4882–4895. e4886. 10.1016/j.cub.2020.09.043 33065005 PMC8182743

[B39] Shannon P. Markiel A. Ozier O. Baliga N. S. Wang J. T. Ramage D. (2003). Cytoscape: A Software Environment for Integrated Models of Biomolecular Interaction Networks. Genome Res. 13, 2498–2504. 10.1101/gr.1239303 14597658 PMC403769

[B40] Shao Z. Ni L. Hu S. Xu T. Meftah Z. Yu Z. (2021). RNA-Binding Protein HuR Suppresses Senescence through Atg7 Mediated Autophagy Activation in Diabetic Intervertebral Disc Degeneration. Cell. Prolif. 54, e12975. 10.1111/cpr.12975 33372336 PMC7848958

[B41] Shrestha M. M. Lim C.-Y. Bi X. Robinson R. C. Han W. (2021). Tmod3 Phosphorylation Mediates AMPK-Dependent GLUT4 Plasma Membrane Insertion in Myoblasts. Front. Endocrinol. 12, 653557. 10.3389/fendo.2021.653557 PMC8095187 33959097

[B42] Smolders L. A. Meij B. P. Onis D. Riemers F. M. Bergknut N. Wubbolts R. (2013). Gene Expression Profiling of Early Intervertebral Disc Degeneration Reveals a Down-Regulation of Canonical Wnt Signaling and Caveolin-1 Expression: Implications for Development of Regenerative Strategies. Arthritis Res. Ther. 15, R23. 10.1186/ar4157 23360510 PMC3672710

[B43] Song Y. Lu S. Geng W. Feng X. Luo R. Li G. (2021). Mitochondrial Quality Control in Intervertebral Disc Degeneration. Exp. Mol. Med. 53, 1124–1133. 10.1038/s12276-021-00650-7 34272472 PMC8333068

[B44] Stich S. Jagielski M. Fleischmann A. Meier C. Bussmann P. Kohl B. (2020). Degeneration of Lumbar Intervertebral Discs: Characterization of Anulus Fibrosus Tissue and Cells of Different Degeneration Grades. Int. J. Mol. Sci. 21 (6), 2165. 10.3390/ijms21062165 PMC7139657 32245213

[B45] Subramanian A. Tamayo P. Mootha V. K. Mukherjee S. Ebert B. L. Gillette M. A. (2005). Gene Set Enrichment Analysis: A Knowledge-Based Approach for Interpreting Genome-Wide Expression Profiles. Proc. Natl. Acad. Sci. U.S.A. 102, 15545–15550. 10.1073/pnas.0506580102 16199517 PMC1239896

[B46] Sun K. Jing X. Guo J. Yao X. Guo F. (2021). Mitophagy in Degenerative Joint Diseases. Autophagy 17, 2082–2092. 10.1080/15548627.2020.1822097 32967533 PMC8496714

[B47] Sun Z. Liu B. Luo Z.-J. (2020). The Immune Privilege of the Intervertebral Disc: Implications for Intervertebral Disc Degeneration Treatment. Int. J. Med. Sci. 17, 685–692. 10.7150/ijms.42238 32210719 PMC7085207

[B48] Szklarczyk D. Gable A. L. Lyon D. Junge A. Wyder S. Huerta-Cepas J. (2019). STRING V11: Protein-Protein Association Networks with Increased Coverage, Supporting Functional Discovery in Genome-Wide Experimental Datasets. Nucleic Acids Res. 47, D607–D613. 10.1093/nar/gky1131 30476243 PMC6323986

[B49] Tang Z. Hu B. Zang F. Wang J. Zhang X. Chen H. (2019). Nrf2 Drives Oxidative Stress-Induced Autophagy in Nucleus Pulposus Cells via a Keap1/Nrf2/p62 Feedback Loop to Protect Intervertebral Disc from Degeneration. Cell. Death Dis. 10, 510. 10.1038/s41419-019-1701-3 31263165 PMC6602960

[B50] Taracha-Wisniewska A. Kotarba G. Dworkin S. Wilanowski T. (2020). Recent Discoveries on the Involvement of Krüppel-Like Factor 4 in the Most Common Cancer Types. Int. J. Mol. Sci. 21 (22), 8843. 10.3390/ijms21228843 PMC7700188 33266506

[B51] Tollenaere M. A. X. Tiedje C. Rasmussen S. Nielsen J. C. Vind A. C. Blasius M. (2019). GIGYF1/2-Driven Cooperation between ZNF598 and TTP in Posttranscriptional Regulation of Inflammatory Signaling. Cell. Rep. 26, 3511–3521. e3514. 10.1016/j.celrep.2019.03.006 30917308

[B52] Tonomura H. Nagae M. Takatori R. Ishibashi H. Itsuji T. Takahashi K. (2020). The Potential Role of Hepatocyte Growth Factor in Degenerative Disorders of the Synovial Joint and Spine. Int. J. Mol. Sci. 21 (22), 8717. 10.3390/ijms21228717 PMC7698933 33218127

[B53] Wan Z.-Y. Song F. Sun Z. Chen Y.-F. Zhang W.-L. Samartzis D. (2014). Aberrantly Expressed Long Noncoding RNAs in Human Intervertebral Disc Degeneration: A Microarray Related Study. Arthritis Res. Ther. 16, 465. 10.1186/s13075-014-0465-5 25280944 PMC4201740

[B54] Wang H. Liu W. Yu B. Yu X. Chen B. (2020a). Identification of Key Modules and Hub Genes of Annulus Fibrosus in Intervertebral Disc Degeneration. Front. Genet. 11, 596174. 10.3389/fgene.2020.596174 33584795 PMC7875098

[B55] Wang H.-Q. Yu X.-D. Liu Z.-H. Cheng X. Samartzis D. Jia L.-T. (2011). Deregulated miR-155 Promotes Fas-Mediated Apoptosis in Human Intervertebral Disc Degeneration by Targeting FADD and Caspase-3. J. Pathol. 225, 232–242. 10.1002/path.2931 21706480

[B56] Wang J. Hu J. Chen X. Huang C. Lin J. Shao Z. (2019a). BRD4 Inhibition Regulates MAPK, NF‐κB Signals, and Autophagy to Suppress MMP‐13 Expression in Diabetic Intervertebral Disc Degeneration. FASEB J. 33, 11555–11566. 10.1096/fj.201900703r 31331201

[B57] Wang R. Xu C. Zhong H. Hu B. Wei L. Liu N. (2020b). Inflammatory‐Sensitive CHI3L1 Protects Nucleus Pulposus via AKT3 Signaling during Intervertebral Disc Degeneration. FASEB J. 34, 3554–3569. 10.1096/fj.201902096r 31997395

[B58] Wang W. Li A. Han X. Wang Q. Guo J. Wu Y. (2020c). DEPDC1 Up‐regulates RAS Expression to Inhibit Autophagy in Lung Adenocarcinoma Cells. J. . Mol. Med. 24, 13303–13313. 10.1111/jcmm.15947 PMC7701574 33021072

[B59] Wang X. Sun J. Tan J. Fang P. Chen J. Yuan W. (2019b). Effect of sIL-13Rα2-Fc on the Progression of Rat Tail Intervertebral Disc Degeneration. J. Orthop. Surg. Res. 14, 386. 10.1186/s13018-019-1361-0 31775818 PMC6880576

[B60] Wang Y. Dai G. Li L. Liu L. Jiang L. Li S. (2019c). Transcriptome Signatures Reveal Candidate Key Genes in the Whole Blood of Patients with Lumbar Disc Prolapse. Exp. Ther. Med. 18, 4591–4602. 10.3892/etm.2019.8137 31777557 PMC6862187

[B61] Wang Y. Dai G. Jiang L. Liao S. Xia J. (2021). Microarray Analysis Reveals an Inflammatory Transcriptomic Signature in Peripheral Blood for Sciatica. BMC Neurol. 21, 50. 10.1186/s12883-021-02078-y 33535986 PMC7856817

[B62] Wu C. Ge J. Yang M. Yan Q. Wang Y. Yu H. (2021). Resveratrol Protects Human Nucleus Pulposus Cells from Degeneration by Blocking IL-6/JAK/STAT3 Pathway. Eur. J. Med. Res. 26, 81. 10.1186/s40001-021-00555-1 34321087 PMC8320225

[B63] Xiang Q. Kang L. Wang J. Liao Z. Song Y. Zhao K. (2020). CircRNA-CIDN Mitigated Compression Loading-Induced Damage in Human Nucleus Pulposus Cells via miR-34a-5p/SIRT1 axis. EBioMedicine 53, 102679. 10.1016/j.ebiom.2020.102679 32114390 PMC7044714

[B64] Xu C. Luo S. Wei L. Wu H. Gu W. Zhou W. (2021). Integrated Transcriptome and Proteome Analyses Identify Novel Regulatory Network of Nucleus Pulposus Cells in Intervertebral Disc Degeneration. BMC Med. Genomics 14, 40. 10.1186/s12920-021-00889-z 33536009 PMC7860219

[B65] Xu S. Feng Y. Zhao S. (2019). Proteins with Evolutionarily Hypervariable Domains are Associated with Immune Response and Better Survival of Basal-Like Breast Cancer Patients. Comput. Struct. Biotechnol. J. 17, 430–440. 10.1016/j.csbj.2019.03.008 30996822 PMC6451114

[B66] Yang Y. Luo H. Zhou C. Zhang R. Liu S. Zhu X. (2019a). Regulation of Capillary Tubules and Lipid Formation in Vascular Endothelial Cells and Macrophages via Extracellular Vesicle-Mediated microRNA-4306 Transfer. J. Int. Med. Res. 47, 453–469. 10.1177/0300060518809255 30477383 PMC6384455

[B67] Yang Y. Zhang B. Nakata M. Nakae J. Mori M. Yada T. (2019b). Islet β-cell-produced NUCB2/nesfatin-1 Maintains Insulin Secretion and Glycemia along with Suppressing UCP-2 in β-Cells. J. Physiol. Sci. 69, 733–739. 10.1007/s12576-019-00689-2 31228099 PMC10717817

[B68] Yu G. (2020). Gene Ontology Semantic Similarity Analysis Using GOSemSim. Methods Mol. Biol. 2117, 207–215. 10.1007/978-1-0716-0301-7_11 31960380

[B69] Yu G. Wang L.-G. Han Y. He Q.-Y. (2012). clusterProfiler: An R Package for Comparing Biological Themes Among Gene Clusters. OMICS 16, 284–287. 10.1089/omi.2011.0118 22455463 PMC3339379

[B70] Zhang B. Sun M. Wang J. Ma C. Hao T. Liu G. (2019). MiR-671 Ameliorates the Progression of Osteoarthritis *In Vitro* and *In Vivo* . Pathol. Res. Pract. 215, 152423. 10.1016/j.prp.2019.04.015 31085006

[B71] Zhang G. Luo Y. Li G. Wang L. Na D. Wu X. (2014). DHRSX, a Novel Non-Classical Secretory Protein Associated with Starvation Induced Autophagy. Int. J. Med. Sci. 11, 962–970. 10.7150/ijms.9529 25076851 PMC4113589

[B72] Zhang H. Zhang M. Meng L. Guo M. Piao M. Huang Z. (2020a). Investigation of Key miRNAs and Their Target Genes Involved in Cell Apoptosis during Intervertebral Disc Degeneration Development Using Bioinformatics Methods. J. Neurosurg. Sci. 66 (2), 125–132. 10.23736/S0390-5616.20.04773-6 32031354

[B73] Zhang J. Hu S. Ding R. Yuan J. Jia J. Wu T. (2021). CircSNHG5 Sponges Mir-495-3p and Modulates CITED2 to Protect Cartilage Endplate from Degradation. Front. Cell. Dev. Biol. 9, 668715. 10.3389/fcell.2021.668715 34277611 PMC8281349

[B74] Zhang J. Zhang J. Zhang Y. Liu W. Ni W. Huang X. (2020b). Mesenchymal Stem Cells‐Derived Exosomes Ameliorate Intervertebral Disc Degeneration through Inhibiting Pyroptosis. J. . Mol. Med. 24, 11742–11754. 10.1111/jcmm.15784 PMC7579702 32860495

